# Diversity-oriented synthesis of analogues of the novel macrocyclic peptide FR-225497 through late stage functionalization

**DOI:** 10.3762/bjoc.11.270

**Published:** 2015-12-08

**Authors:** Jyotiprasad Mukherjee, Suman Sil, Shital Kumar Chattopadhyay

**Affiliations:** 1 Department of Chemistry, University of Kalyani, Kalyani - 741235, West Bengal, India, Fax: +91+33+25828282

**Keywords:** cross metathesis, cyclic peptides, diversity oriented synthesis, macrocycle

## Abstract

A concise synthetic approach to a class of biologically interesting cyclic tetrapeptides is reported which involves a late-stage functionalization of a macrocyclic scaffold through cross metathesis in an attempt to create diversity. The utility of this protocol is demonstrated through the preparation of three structural analogues of the important naturally occurring histone deacetylase inhibitor FR-225497.

## Introduction

**Figure 1:**
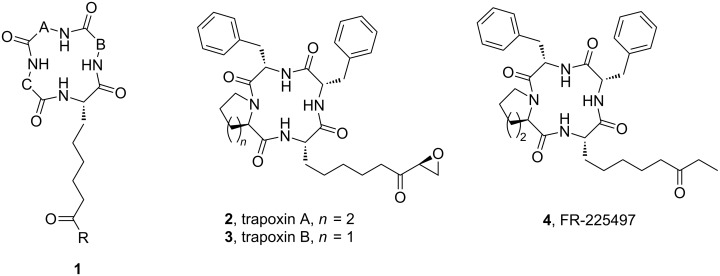
Structure of FR-225497 (**4**) and related cyclic tetrapeptides.

Diversity-oriented synthesis (DOS) has been established as an important paradigm in drug discovery [1–7]. Although the major focus is on the synthesis of small molecular libraries [8], macrocyclic compounds are currently expanding the medicinal chemistry space [9–10]. Macrocyclic natural products [11] with significant levels of biological activity, and their analogues [12] are therefore receiving attention as targets for diversity-oriented synthesis. A class of cyclic tetra peptides of the general structure **1** (Figure 1) displays important histone deacetylase inhibition property relevant to drug design against a number of diseases ranging from antifungal, antimicrobial to arrest of proliferation of several cell types of epithelial and hematological origin [13]. The compounds possess three distinct regions such as a surface recognition head group, a spacer group and a metal-binding end group which fold to provide suitable conformation necessary for their biological activity [14]. For example, compounds **2** and **4** possess identical head groups and the same spacer region; but differ in their end groups. Although there are excellent synthetic approaches to nearly all of such known targets [15], the development of a simple diversity-oriented approach suitable for modification of the compounds remains desirable.

## Results and Discussion

**Figure 2:**
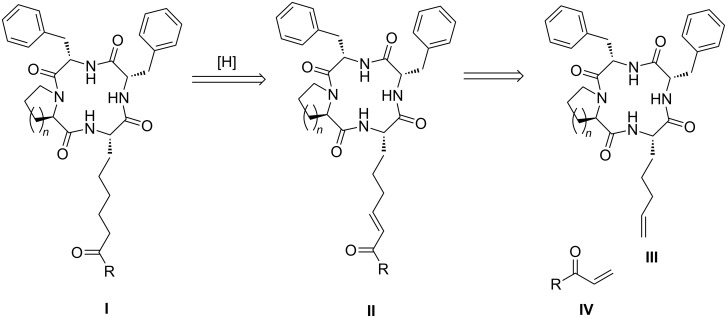
Synthetic planning for the design of analogues of **4**.

We thought that a cross metathesis reaction [16–17] on a macrocyclic template would constitute a direct approach for grafting the pendant alkyl chain of **1**, as demonstrated retrosynthetically in Figure 2. Our initial focus was on the preparation of analogues of the macrocyclic compound **4** through variation of the end groups as it has been shown [18] to have potent activities in the antiproliferation assays with human Jurkat and HT-29 tumor cell lines. Moreover; it also showed excellent immunosuppressive effects suggesting its use as prophylactic agent in organ transplantation. It contains 2-amino-8-oxo-decanoic acid (Aoda) component whereas the trapoxins **2** and **3** contain a 2-amino-8-oxo-9,10-epoxydecanoic acid (Aoe) as the unusual amino acid. The cyclic tetrapeptide scaffold represented by structure **III** is nearly identical to the compounds **2**–**4**, the difference being in the D-proline/D-pipecolic acid segment. The difference may be responsible for the increased activity of compound **4** compared to **2** or **3** [18].

**Scheme 1:**
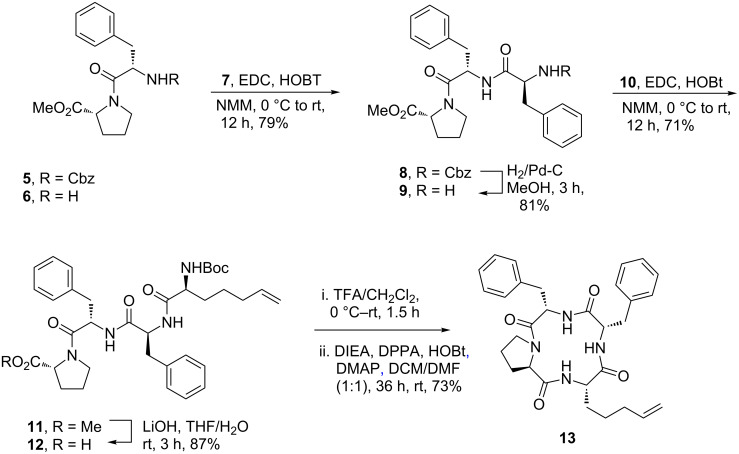
Preparation of the macrocyclic template **13**.

Accommodating some of these common features, we prepared the macrocyclic template **13** (Scheme 1) through the acyclic tetrapeptide Boc-L-Bhag-L-Phe-L-Phe-D-Pro-OMe (**11**) (Bhag = bishomoallylglycine) [19]. The latter was accessed by solution phase peptide coupling between L-Phe-D-Pro-OMe (**6**) (prepared by hydrogenolysis of the corresponding carbamate **5**) and L-Cbz-Phe-OH (**7**) followed by elaboration of the resulting tripeptide **8** via N-terminus deprotection leading to **9** followed by a second peptide coupling of the latter with the known Boc-L-Bhag **10** [20]. The doubly protected tetrapeptide **11** was obtained in an overall yield of 38% in a linear sequence of four steps from **5**. The two termini of **11** were then sequentially deprotected involving ester hydrolysis of **11**→**12** followed by N-deprotection of the latter leading to a crude TFA salt in readiness for a subsequent macrocyclization. The macrocyclization proceeded well under our previously developed conditions [21] to deliver the template **13** in an acceptable yield of 73% over two steps.

**Scheme 2:**
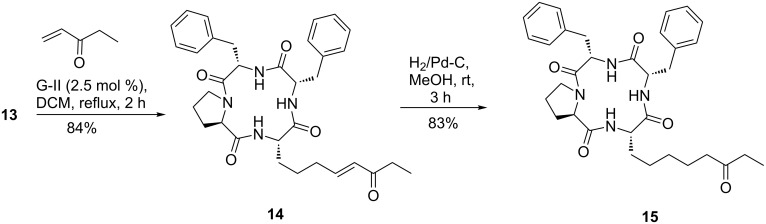
Synthesis of nor-FR225497 (**15**).

We next focused our attention on the crucial cross metathesis reaction of **13.** Attempted CM of **13** (Scheme 2) with ethyl vinyl ketone in the presence of Grubbs’ 1^st^ generation catalyst (G-I) proved to be unsuccessful in our hands under a range of conditions. However, changing to the 2^nd^-generation catalyst [(1,3-bis-(2,4,6-trimethylphenyl)-2-imidazolidinylidene)dichloro(phenylmethylene)(trichlorohexylphosphine)ruthenium, G-II] smoothly effected the desired transformation within a short period of time. Compound **14** was obtained as a single (*E*)-isomer, as expected. Saturation of the double bond in the latter furnished nor-FR225497 (**15**) in an overall yield of ≈70% over two steps from template **13**.

**Scheme 3:**
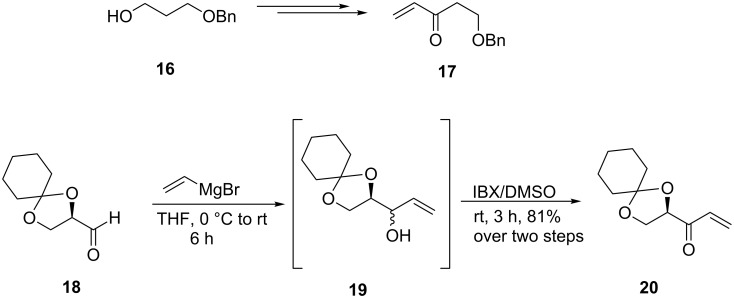
Synthesis of oxygenated ethyl vinyl ketones.

We opted for the CM studies of **13** with olefins **17** and **20** (Scheme 3) for possible incorporation of additional oxygenation pattern in the 9-10 positions of the Aoda fragment in the macrocyclic target in view of the occurrence of such functionalities in compounds **2** and **3** and other related ones [22]. The known olefin **17** [23] was prepared by a straightforward oxidation–vinylation–oxidation sequence on 3-(benzyloxy)propanol (**16**). On the other hand, a vinylation–oxidation sequence on the known aldehyde **18** [24] led to the conjugated olefin **20** through the intermediate **19** (not purified) in good yields.

**Scheme 4:**
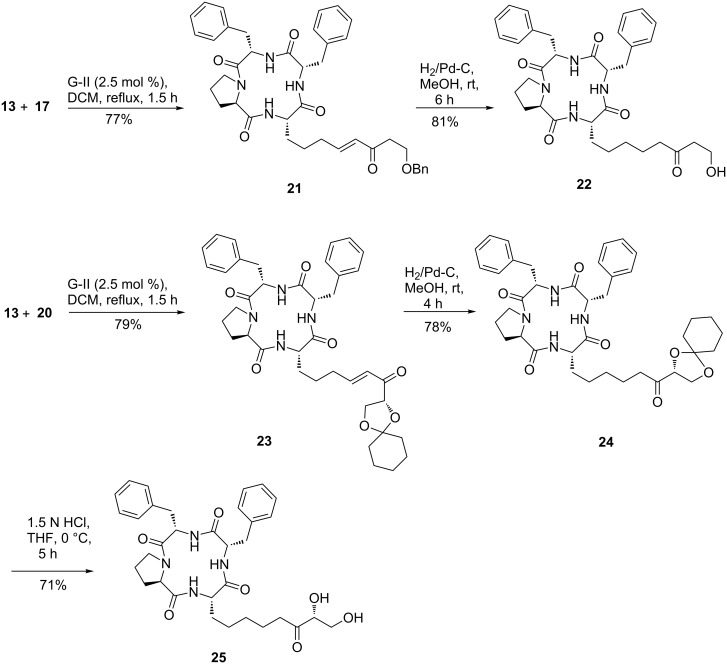
Synthesis of analogues of FR-225497.

Cross metathesis of template **13** with olefin **17** proceeded rapidly under the developed conditions to provide product **21** (Scheme 4) in good yield. Saturation of the double bond in the later with hydrogen also proceeded without events to provide the 10-hydroxylated product **22**. Similarly, CM with olefin **20** proceeded well analogously to provide the macrocyclic product **23**. Compound **23** was further transformed to the C9,C10-dihydroxylated macrocyclic product **25** via two conventional steps viz. saturation of the double bond leading to **24** followed by an acid-mediated deprotection of the dioxolane ring in the latter. The macrocyclic products **15**, **22** and **25** have the nearly same head group and spacer as in the trapoxins and FR-225497 but differ in the end groups. A α-hydroxyketone or a α,ß-dihydroxyketone moiety in the end-region may prove to be beneficial for SAR studies in view of the importance of these functionalities in metal binding.

## Conclusion

In conclusion, we have been able to demonstrate that cross metathesis reaction on a cyclic tetrapeptide template is a viable strategy for the synthesis of a class of macrocyclic natural product-based analogues. The developed protocol may provide scope for judicious manipulation of the spacer region as well as the metal-binding domain attached to a particular surface recognition part present in the class of these compounds since histone deacetylase activity has been correlated to zinc-binding ability of the 8-oxo moiety in some of such compounds [25]. Moreover, the opportunity of incorporation of other functional groups compatible with CM reaction appears possible. The protocol developed is simple and high yielding. This apparently overlooked approach may therefore find application in the assembly of focused library of simplified agents for possible SAR studies.

## Supporting Information

File 1 Experimental details and analytical data of all new compounds as well as copies of their ^1^H and ^13^C NMR spectra.
