# Supplementary material for: Diversity-oriented synthesis of analogues of the novel macrocyclic peptide FR-225497 through late stage functionalization
Source: Beilstein J Org Chem. 2015 Dec 8;11:2487–92. doi: 10.3762/bjoc.11.270 (PMC4685926; doi:10.3762/bjoc.11.270)
Supplement: File 1 — Experimental details and analytical data of all new compounds as well as copies of their 1H and 13C NMR spectra. [file Beilstein_J_Org_Chem-11-2487-s001.pdf]

Supporting Information

for

Diversity-oriented synthesis of analogues of the novel macrocyclic peptide FR-225497 through late stage functionalization

Jyotiprasad Mukherjee, Suman Sil and Shital Kumar Chattopadhyay*

Address: Department of Chemistry, University of Kalyani, Kalyani - 741235, West Bengal, India, Fax: +91+33+25828282

Email: Shital Kumar Chattopadhyay - skchatto@yahoo.com

*Corresponding author

Experimental details and analytical data of all new compounds as well as copies of their ^{1}H and ^{13}C NMR spectra

Content **page**

Experimental procedures ………………………………………………………………………. S2–S10

Copies of ^{1}H and ^{13}C NMR spectra of the reported compounds …………………………..…. S11–S47

Experimental procedures

General: Column chromatography was performed on silica gel, Merck grade 230–400 mesh and neutral alumina. Reactions were monitored by thin-layer chromatography; TLC plates were visualized with UV light, in an iodine chamber, or with vaniline solution, unless noted otherwise. Melting points were recorded in open capillaries and are uncorrected. IR spectra were recorded using KBr disks, chloroform solution or as neat. Chemical shifts (δ) are given from TMS (0 ppm) as internal standard for ^{1}H NMR and ^{13}CDCl$_3$ (77.0 ppm) for ^{13}C NMR. The following abbreviations were used to explain the multiplicities: s = singlet, d = doublet, t = triplet, q = quartet, m = multiplet, dd = double doublet, ddd = doublet of double doublet, dt = doublet of triplet, br = broad, etc. HRMS data were recorded on a Waters XEVO G2 QTOF instrument purchased through DST-PURSE Grant.

Dichloromethane and dimethyl sulfoxide were distilled over calcium hydride under an inert atmosphere. THF, toluene, benzene and ether were freshly distilled under argon from a purple solution of sodium benzophenone ketyl. Unless stated otherwise, all reagents were purchased from commercial sources and used without additional purification.

*(R)-Methyl 1-((S)-2-((S)-2-(benzyloxycarbonylamino)-3-phenylpropanamido)-3-phenylpropanoyl)-pyrrolidine-2-carboxylate (**8**).*

N-Methylmorpholine (450 µL, 4 mmol) was added dropwise to a stirred solution of the amine **6** (1.1 g, 4 mmol), acid **7** (1.2 g, 4 mmol), EDC·HCl (800 mg, 4 mmol) and HOBT (540 mg, 4 mmol) in a mixture of anhydrous DCM and DMF (1:1, 40 mL) over 10 min at 0 ℃ under N_2 atmosphere while stirred. The reaction mixture was then allowed to come to room temperature and stirring was continued for another 12 h before being diluted with DCM (50 mL). The organic extract was washed successively with a saturated aq solution of $NaHCO_3$ (30 mL), HCl (1 N, 30 mL), water (40 mL) and brine solution (40 mL), and then dried over anhydrous $MgSO_4$. It was then filtered and the filtrate was concentrated in vacuo to leave the crude product as a light

yellow viscous solid which was purified by column chromatography over silica gel using a mixture of petroleum ether/ethyl acetate (7:3) as eluent to provide the coupled product **8** (1.70 g, 79%) as a colorless solid: mp 186-188 ℃ (recrystallised from EtOAc-hexane). $[\alpha]_D^{25}$ +26.7 (*c* 1.6, $CHCl_3$); IR (neat) 3360, 3228, 303, 1743, 1712, 1651, 1631, 1234 cm^{-1}; ^1H NMR (400 MHz, $CDCl_3$): δ = 7.37-7.16 (m, 15 H), 6.71 (d, *J* = 7.6 Hz, 1 H), 5.26 (d, *J* = 7.6 Hz, 1 H), 5.08 (s, 2 H), 4.90 (dt, *J* = 5.2, 8.8 Hz, 1 H), 4.46 (q, *J* = 6.8 Hz, 1 H), 4.30 (dd, *J* = 4, 8 Hz, 1 H), 3.69 (s, 3 H), 3.47-3.45 (m, 1 H), 3.11-3.08 (m, 1 H), 3.04-2.98 (m, 2 H), 2.93-2.88 (m, 1 H), 1.94-1.84 (m, 4 H), 1.55-1.52 (m, 1 H); ^{13}C NMR (100 MHz, $CDCl_3$): δ = 172.1, 170.5, 169.7, 155.9, 136.9, 136.4, 136.2, 129.5, 129.3, 128.5, 128.5, 128.4, 128.1, 128.0, 127.1, 126.9, 66.8, 58.8, 55.8, 52.5, 52.2, 46.9, 39.6, 38.6, 29.0, 24.4; HRMS (QTOF ES+) found *m/z* 558.2615 $(M + H)^+$, $C_{32}H_{36}N_3O_6$ requires 558.2604.

*(R)-Methyl 1-((S)-2-((S)-2-amino-3-phenylpropanamido)-3-phenylpropanoyl)pyrrolidine-2-carboxylate (**9**).*

Pd-C (60 mg) was added to a stirring solution of **8** (550 mg, 1.0 mmol) in dry MeOH (10 mL) and the resulting heterogeneous mixture was then vigorously stirred for 3 h at room temperature under hydrogen atmosphere. The reaction mixture was then filtered through celite, the filter cake was washed with methanol (10 mL) and the combined filtrate was concentrated in vacuo. The residue was purified by column chromatography over silica gel using a mixture of ethyl acetate in hexane (1:1) as eluent to provide the amine **9** (342 mg, 81%) as a colorless gummy solid: $[\alpha]_D^{25}$ +4.89 (c 1.4, $CHCl_3$); IR (neat) 3363, 3062, 2953, 1743, 1644, 1497, 1451, 1197, 1174 cm^{-1}; ^1H NMR (400 MHz, $CDCl_3$) δ = 7.97 (d, *J* = 8.8 Hz, 1 H), 7.32-7.16 (m, 10 H), 7.07 (d, *J* = 6.8 Hz, 1 H), 5.00 (dt, *J* = 6.4, 2.4 Hz, 1 H), 4.31 (dd, *J* = 3.6, 7.6 Hz, 1 H), 3.6 (s, 3 H), 3.61-3.57 (m, 2 H), 2.99-2.96 (m, 2 H), 2.80 (dd, *J* = 4.4, 2.8 Hz, 1 H), 2.61 (dd, *J* = 9.6, 13.2 Hz, 1 H), 1.94-1.79 (m, 4 H), 1.17 (brs, 2 H); ^{13}C NMR (100 MHz, $CDCl_3$) δ = 173.8, 172.3, 169.9, 137.8, 136.4, 129.5, 129.3, 128.6, 128.4, 127.0, 126.8, 58.7, 56.3, 52.3, 51.8, 46.9, 40.8, 39.5, 28.9, 24.4; HRMS (QTOF ES+) found *m/z* 424.2225 $(M + H)^+$, $C_{24}H_{30}N_3O_4$ requires 424.2236.

*(R)-methyl 1-((6S,9S,12S)-9,12-dibenzyl-2,2-dimethyl-4,7,10-trioxo-6-(pent-4-enyl)-3-oxa-5,8,11-triazatridecan-13-oyl)pyrrolidine-2-carboxylate (**11**).*

Compound **11** was prepared from amine **9** and acid **10** following exactly the procedure described for **8** and the product was obtained as a colorless foam (1.86 g, 71%): $[\alpha]_D^{25}$ +14.0 (*c* 0.50, $CHCl_3$); IR ($CHCl_3$) 3287, 2930, 1751, 1699, 1638, 1523, 1452, 1171 cm^{-1}; ^1H NMR ($CDCl_3$, 400 MHz) δ = 7.30-7.15 (m, 10 H), 6.70 (d, *J* = 7.6 Hz, 1 H), 6.62 (d, *J* = 7.6 Hz, 1 H), 5.78-5.69 (m, 1H), 5.01-4.97(m, 1 H), 4.96-4.95 (m, 1 H), 4.90-4.84 (m, 1 H), 4.68-4.63 (m, 1 H), 4.30 (dd, *J* = 4.0, 8.0 Hz, 1 H), 4.08-4.03 (m, 1 H), 3.71 (s, 3 H), 3.48-3.43 (m, 1 H), 3.10 (dd, *J* = 6.4, 14.0 Hz, 1 H), 3.04-3.00 (m, 1H), 2.99-2.94 (m, 1 H), 2.90(dd, *J* = 9.6, 12.8 Hz, 1 H), 2.67-2.61 (m, 1 H), 2.05-2.01 (m, 2 H), 1.91-1.77 (m, 3 H), 1.75-1.68 (m, 4 H), 1.54-1.49 (m, 2 H), 1.44 (s, 9 H); ^{13}C NMR ($CDCl_3$, 100 MHz) δ = 72.1, 171.9, 169.9, 169.4, 155.6, 138.1, 136.2, 136.1, 129.5, 129.4, 128.5, 128.4, 127.0, 126.9, 115.0, 79.9, 58.7, 54.5, 53.9, 52.4, 52.2, 46.8, 39.6, 38.4, 33.3, 32.1, 28.9, 28.3, 24.7, 24.3; HRMS (QTOF ES+) found *m/z* 671.3420 $(M + Na)^+$, $C_{36}H_{48}N_4NaO_7$ requires 671.3421.

*(R)-1-((6S,9S,12S)-9,12-Dibenzyl-2,2-dimethyl-4,7,10-trioxo-6-(pent-4-enyl)-3-oxa-5,8,11-triazatridecan-13-oyl)pyrrolidine-2-carboxylic acid (**12**).*

Solid $LiOH{\cdot}H_2O$ (210 mg, 5 mmol) was added to a stirring solution of the ester **11** (1.3 g, 2 mmol) in THF-H_2O (4:1, 10 mL) and the reaction mixture was stirred for 3 h at room temperature. The resulting mixture was then acidified to pH 2 with 2 N HCl at 0 °C and the mixture was concentrated under reduced pressure to leave a crude mass which was taken in ethyl acetate (80 mL). The organic part was washed successively with water (30 mL), brine (25 mL) and then dried over $MgSO_4$. It was then filtered, and the filtrate was concentrated in vacuo to leave the crude product which was purified by column chromatography over silica gel using a mixture of chloroform/methanol (19:1) as eluent to provide the acid (1.1 g, 87%) as a colorless solid: mp 125-127 °C (recrystallised from $CHCl_3$). $[\alpha]_D^{25}$ +2.3 (*c* 1.0, $CHCl_3$); IR (neat) 3294, 3030, 2931, 1716, 1638, 1498, 1455 cm^{-1}; ^1H NMR ($CDCl_3$, 400 MHz) δ = 8.56 (d, *J* = 8.0 Hz, 1 H), 7.26-7.18 (m, 6 H), 7.16-7.13 (m, 2 H), 7.10-7.06 (m, 1 H), 7.01-6.94 (m, 2 H), 5.77-5.67 (m, 1 H), 5.05 (brd, *J* = 5.6 Hz, 2 H), 4.99-4.91 (m, 3 H), 4.23

(dd, J = 4.0, 7.6 Hz, 1 H), 4.13 (brd, J = 3.2 Hz, 1 H), 3.61-3.59 (m, 1 H), 3.18-3.13 (m, 1 H), 3.05 (dd, J = 4.0, 13.6 Hz, 2 H), 2.81 (dd, J = 6.0, 13.6 Hz, 2 H), 2.00-1.93 (m, 5 H), 1.70-1.68 (m, 1 H), 1.55-1.48 (m, 2 H), 1.43 (s, 9 H), 1.38-1.36 (m, 3 H); ^{13}C NMR ($CDCl_3$, 100 MHz) δ = 173.2 (-CO_2H), 172.5 (-CON-), 170.7(-CON-) , 170.0(-CON-), 155.5(-N-$\underline{C}$O-O-CMe_3), 138.1 (Ar-C), 136.2 (Ar-C), 135.6 ($\underline{C}H{=}CH_2$), 129.8 (Ar-CH), 129.5(Ar-CH), 128.5(Ar-CH), 128.2(Ar-CH), 127.1(Ar-CH), 126.7(Ar-CH), 115.0($CH{=}\underline{C}H_2$) , 80.1(-N-CO-O-$\underline{C}Me_3$), 59.0 (-N-$\underline{C}$H-CO_2H), 54.5(-NH-$\underline{C}$H-CO), 53.6(-NH-$\underline{C}$H-CO), 52.2(-NH-$\underline{C}$H-CO), 47.5(-N-$\underline{C}H_2$-CH_2),, 39.5 ($PhCH_2$), 38.6 ($PhCH_2$), 33.3 (-$\underline{C}H_2$-C=C-), 32.5 (NHBoc-CH-$\underline{C}H_2$), 28.8 (proline CH_2), 28.3 (-C$\underline{Me_3}$), 24.7 (two signals, proline CH_2 + homoallylic $\underline{C}H_2$). HRMS (QTOF ES+) found m/z 657.3272 $(M + Na)^+$, $C_{35}H_{46}N_4NaO_7$ requires 657.3264.

*(3S,6S,9S,14aR)-6,9-Dibenzyl-3-(pent-4-enyl)decahydropyrrolo[1,2-a][1,4,7,10]tetraazacyclododecine-1,4,7,10-tetraone (**13**).*

A solution of **12** (504 mg, 0.79 mmol) in DCM (20 mL) was cooled to 0 ℃ under nitrogen atmosphere and TFA (8.5 mL) was added dropwise to it. The resulting solution was stirred for 1.5 h at the same temperature and then concentrated in vacuo to leave the crude TFA salt as a yellowish solid which was used as such at the next step. DPPA (270 µl) followed by DIEA (300 µL) were added sequentially to a solution of HOBT (160 mg) and DMAP (48 mg) in a mixture of anhydrous DCM (35 mL) and DMF (35 mL) at room temperature under nitrogen atmosphere and the resulting mixture was stirred for 15 min. A solution of the crude TFA-salt in anhydrous DMF (10 mL) was added drop wise to the aforesaid solution at the same temperature. The resulting mixture was allowed to stir for 36 h at room temperature before being poured into aqueous HCl (1N, 60 mL) at 0℃. It was then extracted with DCM (2 × 50 mL) and the combined extract was washed successively with HCl (3N, 40 mL), saturated aq solution of $NaHCO_3$ (70 mL), water (60 mL) and brine (70 mL). It was then dried over $MgSO_4$, filtered and the filtrate was concentrated in vacuo to leave the crude product as a yellowish solid which was purified by column chromatography over silica gel using petroleum ether/ethyl acetate (4:6) as eluent to afford the cyclopeptide product **13** (295 mg, 73%) as a colorless solid: mp 208-210 ℃ (recrystallised

from EtOAc-hexane). $[\alpha]_D^{25}$ +43.1 (*c* 0.75, $CHCl_3$); IR (neat) 3330, 3062, 2927, 1654, 1637, 1529, 1440, 1245 cm^{-1}; ^1H NMR ($CDCl_3$, 400 MHz) δ = 7.84 (brs, 1 H), 7.39 (d, *J* = 6.0 Hz, 1 H), 7.30-7.13 (m, 10 H), 6.95 (brd, 1 H), 5.77-5.67 (m, 1 H), 4.95 (dd, *J* = 1.6, 16.8 Hz, 1 H), 4.90 (dd, *J* = 1.6, 10.0 Hz, 1 H), 4.62 (quin, *J* = 5.2 Hz, 1 H), 4.40 (brm, 1 H), 4.22 (dd, *J* = 3.2, 7.2 Hz, 1 H), 4.15-4.09 (m, 1 H), 3.28-3.25 (m, 1 H), 3.22-3.06 (m, 2 H), 3.02 (dd, *J* = 5.2, 12.8 Hz, 1 H), 2.70 (q, *J* = 8.0 Hz, 1 H), 2.09-2.00 (m, 5 H), 1.92-1.81 (m, 1 H), 1.76-1.67 (m, 2 H), 1.50-1.47 (m, 1 H), 1.36-1.30 (m, 2 H); ^{13}C NMR ($CDCl_3$, 100 MHz) δ = 173.1, 172.4, 171.6, 170.9, 138.1, 137.5, 136.2, 129.3, 129.3, 128.6, 128.4, 127.2, 126.7, 115.0, 60.8, 56.9, 54.3, 53.8, 46.8, 37.5, 36.7, 30.0, 29.7, 27.7, 25.2, 24.5; HRMS (QTOF ES+) found *m/z* 539.2645 $(M + Na)^+$, $C_{30}H_{36}N_4NaO_4$ requires 539.2634.

*(3S,6S,9S,14aR)-6,9-Dibenzyl-3-((E)-6-oxooct-4-enyl)decahydropyrrolo[1,2-a][1,4,7,10]tetraazacyclododecine-1,4,7,10-tetraone (**14**).*

Grubbs' second generation catalyst (G-II, 10 mg, 0.012 mmol, 2.5 mol %) was added to a stirred solution of the cyclopeptide **13** (256 mg, 0.5 mmol) and ethyl vinyl ketone (105 mg, 125 µL, 1.25 mmol) in dry and degassed DCM (10 mL) and the resulting reaction mixture was heated to reflux for 2 h. It was allowed to cool to room temperature and then concentrated in vacuo. The residue was purified by chromatography over silica gel using a mixture of ethyl acetate in hexane (1:1) to provide the coupled product **14** (240 mg, 84%) as a colorless viscous liquid: $[\alpha]_D^{25}$ +17.6 (*c* 1.0, $CHCl_3$); IR ($CHCl_3$) 3327, 2925, 2855, 1662, 1634, 1535, 1454, 1355, 1276, 1244 cm^{-1}; ^1H NMR ($CDCl_3$, 400 MHz) δ = 7.79 (brs, 1 H), 7.41 (d, *J* = 5.6 Hz, 1 H), 7.34-7.25 (m, 6 H), 7.22-7.14 (m, 4 H), 7.00 (brs, 1 H), 6.74 (td, *J* = 6.8, 16.0 Hz, 1 H), 6.05 (d, *J* = 16.0 Hz, 1 H), 4.62-4.60 (m, 1 H), 4.29 (brs, 1 H), 4.21 (dd, *J* = 3.6, 7.6 Hz, 1 H), 4.12 (brs, 1 H), 3.39 (brs, 1 H), 3.24-3.15 (m, 2 H), 3.04 (d, *J* = 9.6 Hz, 1 H), 3.02 (dd, *J* = 5.6, 12.8 Hz, 1 H), 2.72-2.69 (m, 1 H), 2.51 (q, *J* = 7.2 Hz, 2 H), 2.15 (quin, *J* = 7.2 Hz, 1 H), 2.02 (dt, *J* = 5.6, 12.0 Hz, 1H), 1.88-1.83 (m, 1 H), 1.77-1.71 (m, 4 H), 1.53-1.51 (m, 1 H), 1.38 (t, *J* = 8.0 Hz, 2 H), 1.06 (t, *J* = 7.2 Hz, 3 H); ^{13}C NMR ($CDCl_3$, 100 MHz) δ = 201.4, 172.9, 172.4, 171.9, 171.0, 146.1, 137.5, 135.9, 130.3, 129.3, 129.0, 128.6, 128.5, 127.3, 126.8, 60.9, 57.0, 54.1, 53.8, 47.0,

37.5, 36.5, 33.3, 31.8, 30.0, 29.7, 28.1, 24.4, 8.1; HRMS (QTOF ES+) found *m/z* 595.2880 $(M + Na)^+$, $C_{33}H_{40}N_4NaO_5$ requires 595.2896.

*(3S,6S,9S,14aR)-6,9-Dibenzyl-3-(6-oxooctyl)decahydropyrrolo[1,2-a][1,4,7,10]tetraazacyclododecine-1,4,7,10-tetraone (**15**).*

The compound was prepared from **14** following the procedure described for **9** and the product was obtained as a colorless foam (117 mg, 83% from 143 mg of **14**): $[\alpha]_D^{25}$ +10.2 (*c* 0.5, $CHCl_3$); IR ($CHCl_3$) 3348, 2936, 2878, 1657, 1637, 1535, 1454, 1086, 1024 cm^{-1}; ^1H NMR ($CDCl_3$, 400 MHz) δ = 7.83 (s, 1 H), 7.41-7.17 (m, 10 H), 7.04-6.96 (m, 1 H), 4.65-4.63 (m, 1 H), 4.39-4.33 (m, 1 H), 4.24-4.16 (m, 2 H), 3.41-3.29 (m, 1 H), 3.20 (d, *J* = 6.8 Hz, 2 H), 3.13 (t, *J* = 12.0 Hz, 1 H), 3.03 (dd, *J* = 4.8, 12.8 Hz, 1 H), 2.74 (m, 1 H), 2.37 (q, *J* = 7.2 Hz, 2 H), 2.33 (t, *J* = 7.2 Hz, 2 H), 2.03 (q, *J* = 5.6 Hz, 2 H), 1.89-1.78 (m, 3 H), 1.73-1.67 (m, 2 H), 1.51-1.43 (m, 3 H), 1.25-1.20 (m, 3 H), 1.02 (t, *J* = 7.2 Hz, 3 H); ^{13}C NMR ($CDCl_3$, 100 MHz) δ = 211.9, 173.0, 172.1, 171.9, 170.8, 137.5, 136.2, 129.4, 129.3, 128.6, 128.5, 127.2, 126.7, 60.8, 56.8, 54.2, 53.8, 46.9, 42.2, 37.6, 36.7, 35.9, 30.4, 28.7, 27.9, 25.6, 24.6, 23.5, 7.8; HRMS (QTOF ES+) found *m/z* 575.3240 $(M + H)^+$, $C_{33}H_{43}N_4O_5$ requires 575.3233.

*(R)-1-(1,4-Dioxaspiro[4.5]decan-2-yl)prop-2-en-1-one (**20**).*

Vinylmagnesium bromide (2 M in THF, 4.0 mL, 8 mmol) was added dropwise to a stirred solution of (*R*)-2,3-*O*-cyclohexylideneglyceraldehyde (**18**, 1.0 g, 6 mmol) in anhydrous THF (5 mL) at 0 ℃ under a nitrogen atmosphere and the reaction mixture was stirred for 30 min at the same temperature. It was then allowed to come to room temperature and stirred for 5 h. It was cooled back to 0 ℃ and then quenched with drop wise addition of saturated aq NH_4Cl (5 mL). The volatiles were removed under reduced pressure and the residue was diluted with dichloromethane (80 mL). The organic solution was washed successively with aqueous NH_4Cl solution (10%, 2 × 50 mL), water (100 mL) and brine (100 mL), and then dried over anhydrous $MgSO_4$. It was filtered, and the filtrate was concentrated under reduced pressure to provide the crude allylic alcohol **19** which was used without purification in the next step. IBX (1.9 g, 8 mmol) was added in one portion

to a stirred solution of the crude allylic alcohol in dimethyl sulfoxide (12 mL) at room temperature and the stirring was continued for 3 h. The reaction mixture was then poured into a mixture (1:1) of saturated aq $Na_2S_2O_3$solution and saturated aq $NaHCO_3$ (60 mL). The aqueous phase was extracted with DCM (2 × 50 mL) and the combined organic layer was washed successively with water (2 × 50 mL) and brine (50 mL). It was then dried over anhydrous $MgSO_4$, filtered and the filtrate was concentrated in vacuo to provide the crude product which was purified by chromatography over silica gel using a mixture (1:9) of ethyl acetate in petroleum ether to give the α,β unsaturated ketone **20** (948 mg, 81%) as a colorless viscous liquid: $[\alpha]_D^{25}$ +35.5 (*c* 0.75, $CHCl_3$); IR ($CHCl_3$) 3374, 2977, 1744, 1717, 1366, 1165 cm^{-1}; ^1H NMR ($CDCl_3$, 400 MHz) δ = 6.83 (dd, *J* = 10.4, 17.6 Hz, 1 H), 6.43 (dd, *J* = 1.6, 17.6 Hz, 1 H), 5.84 (dd, *J* = 1.6, 10.4 Hz, 1 H), 4.62 (dd, *J* = 5.6, 7.2 Hz, 1 H), 4.22 (dd, *J* = 7.6, 8.4 Hz, 1 H), 4.08 (dd, *J* = 5.6, 8.4 Hz, 1 H), 1.66-1.59 (m, 8 H), 1.43 (s, 2 H); ^{13}C NMR ($CDCl_3$, 100 MHz) δ = 198.8, 131.3, 130.3, 111.8, 79.2, 66.1, 35.5, 34.7, 25.0, 23.9, 23.8; HRMS (QTOF ES+) found *m/z* 219.0978 $(M + Na)^+$, $C_{11}H_{16}NaO_3$ requires 219.0997.

*(3S,6S,9S,14aR)-6,9-Dibenzyl-3-((E)-8-(benzyloxy)-6-oxooct-4-enyl)decahydropyrrolo[1,2-a][1,4,7,10]tetraazacyclododecine-1,4,7,10-tetraone (**21**).*

This was prepared following exactly the procedure described for **14** and the product was obtained as a colorless foam (262 mg, 77%): $[\alpha]_D^{25}$ +18.6 (*c* 2.5, $CHCl_3$); IR ($CHCl_3$): 3331, 2925, 2855, 1650, 1537, 1497, 1454 cm^{-1}; ^1H NMR ($CDCl_3$, 400 MHz) δ = 7.83 (s, 1 H), 7.52-7.18 (m, 15 H), 7.00 (s, 1 H), 6.77 (td, *J* = 6.8, 16 Hz, 1 H), 6.06 (d, *J* = 16.0 Hz, 1 H), 4.68-4.60 (m, 1 H), 4.50 (s, 2 H), 4.30 (brs, 1 H), 4.21-4.20 (m, 1 H), 4.12 (m, 1 H), 3.75 (t, *J* = 6.4 Hz, 2 H), 3.40 (m, 1 H), 3.20-3.00 (m, 4 H), 2.81 (t, *J* = 6.4 Hz, 2 H), 2.69 (m, 1 H), 2.15 (quin, *J* = 7.2 Hz, 2 H), 1.86-1.65 (m, 7 H), 1.39-1.25 (m, 2 H); ^{13}C NMR ($CDCl_3$, 100 MHz) δ = 198.5, 172.8, 172.4, 171.9, 171.0, 147.2, 138.2, 137.5, 136.0, 130.7, 129.3, 129.0, 128.7, 128.5, 128.4, 127.7, 127.6, 127.3, 126.7, 73.2, 65.4, 60.9, 57.1, 54.1, 53.8, 47.0, 40.1, 37.6, 36.6, 31.9, 30.0, 28.1, 24.5, 24.4; HRMS (QTOF ES+) found *m/z* 701.3309 $(M + Na)^+$, $C_{40}H_{46}N_4NaO_6$ requires 701.3315.

*(3S,6S,9S,14aR)-6,9-Dibenzyl-3-(8-hydroxy-6-oxooctyl)decahydropyrrolo[1,2-a][1,4,7,10]tetraazacyclododecine-1,4,7,10-tetraone (**22**).*

The compound was prepared from **21** following exactly the procedure described for **9** but using 6 h and the product was obtained as a colorless foam (121 mg, 81%): $[\alpha]_D^{25}$ +9.6 (*c* 0.5, $CHCl_3$); IR ($CHCl_3$) 3331, 3061, 2929, 2859, 1654, 1637, 1536, 1498 cm^{-1}; ^1H NMR ($CDCl_3$, 400 MHz) δ = 7.85 (brs, 1 H), 7.41 (brs, 1 H), 7.27-7.24 (m, 10 H), 7.06 (brs, 1 H), 4.59 (brs, 1 H), 4.43 (brs, 1 H), 4.27 (brs, 1 H), 4.17-4.11 (m, 1 H), 3.83 (s, 2 H), 3.18-3.04 (m, 5 H), 2.66-2.53 (m, 3 H), 2.39 (brs, 2 H), 2.05 (brs, 2 H), 1.83-1.52 (m, 6 H), 1.26-1.24 (m, 5 H); ^1H NMR (DMSO-d_6, 400 MHz) δ = 7.81 (brs, 1 H), 7.36-7.19 (m, 13 H), 4.56 (s, 2 H),4.34-4.26 (m, 2 H), 4.13 (brs, 1 H), 3.59 (d, *J* = 4.8 Hz, 2 H), 3.40-3.29 (m, 1H, merged with DMSO signal), 2.92 (d, *J* = 12.0 Hz, 2 H), 2.33 (s, 2 H), 2.07-1.96 (m, 3 H), 1.75-1.55 (m, 5 H),1.33-1.09 (brs, 3 H), 1.15-0.99 (m, 5 H). ^{13}C NMR ($CDCl_3$, 100 MHz) δ = 211.7, 173.1, 172.1, 171.6, 170.8, 137.4, 136.2, 129.4, 129.3, 128.6, 128.5, 127.2, 126.7, 60.6, 57.8, 56.6, 54.0, 53.8, 46.8, 44.5, 43.0, 37.5, 36.5, 30.4, 28.3, 27.8, 25.4, 24.5, 23.1; HRMS (QTOF ES+) found *m/z* 613.3031 $(M + Na)^+$, $C_{33}H_{42}N_4NaO_6$ requires 613.3002.

*(3S,6S,9S,14aR)-6,9-Dibenzyl-3-((E)-6-oxo-6-((R)-1,4-dioxaspiro[4.5]decan-2-yl)hex-4-enyl)decahydropyrrolo[1,2-a][1,4,7,10]tetraazacyclododecine-1,4,7,10-tetraone (**23**).*

This was prepared from **13** and **20** following exactly the procedure described for **14** and the product **23** was obtained as a colorless foam (270 mg, 79%): $[\alpha]_D^{25}$ +41.0 (*c* 1.0, $CHCl_3$); IR ($CHCl_3$) 3363, 2978, 2933, 1715, 1652, 1505, 1367, 1164 cm^{-1}; ^1H NMR ($CDCl_3$, 400 MHz) δ = 7.95 (s, 1 H), 7.47 (d, *J* = 6 Hz, 1 H), 7.28-7.00 (m, 11 H), 6.94 (dt, *J* = 15.6, 6.8 Hz, 2 H), 6.50 (d, *J* = 16.0 Hz, 1 H), 4.62-4.56 (m, 1 H), 4.52(t, *J* = 6.8 Hz, 1 H), 4.41-4.36 (m, 1 H), 4.22 (t, *J* = 8.8 Hz, 3 H), 4.00 (dd, *J* = 8.4, 6.0 Hz, 1 H), 3.39-3.29 (m, 1 H), 3.19 (d, *J* = 4.8 Hz, 2 H), 3.14-3.08 (m, 1 H), 3.02 (dd, *J* = 12.8, 5.6 Hz, 2 H), 2.70-2.65 (m, 1 H), 2.18 (quin, *J* = 7.6 Hz, 2 H), 2.04-2.00 (m, 2 H), 1.87-1.53 (m, 10 H), 1.42-1.40 (m, 4 H), 1.30-1.24 (m, 1 H); ^{13}C NMR ($CDCl_3$, 100 MHz) δ = 198.5, 172.8, 172.4, 171.9, 171.0, 149.2, 137.5, 136.0, 129.3, 129.0, 128.6, 128.5, 127.3, 126.7,

125.2, 111.6, 79.2, 66.2, 60.9, 57.2, 54.1, 53.8, 47.0, 37.5, 36.6, 35.5, 34.8, 32.2, 30.0, 28.1, 25.0, 24.6, 24.4, 23.9, 23.8; HRMS (QTOF ES+) found *m/z* 707.3409 $(M + Na)^+$, $C_{39}H_{48}N_4NaO_7$ requires 707.3421.

*(3S,6S,9S,14aR)-6,9-Dibenzyl-3-(6-oxo-6-((R)-1,4-dioxaspiro[4.5]decan-2-yl)hexyl)decahydropyrrolo[1,2-a][1,4,7,10]tetraazacyclododecine-1,4,7,10-tetraone (**24**).*

This was prepared following exactly the procedure described for **9** but using 4 h and the product was obtained as a colorless foam (136 mg, 78%): $[\alpha]_D^{25}$ +27.9 (*c* 0.25, $CHCl_3$); IR ($CHCl_3$) 3373, 2977, 2933, 1732, 1715, 1505, 1367, 1164 cm^{-1}; ^1H NMR ($CDCl_3$, 400 MHz) δ = 7.90-7.75 (m, 1 H), 7.35-7.10 (m, 11 H), 7.09 (s, 1 H), 4.63-4.56 (m, 1 H), 4.37-4.28 (m, 1 H), 4.15-4.13 (m, 1 H), 4.06 (t, *J* = 8.4 Hz, 2 H), 3.84 (dd, *J* = 5.6 Hz, 1 H), 3.24-3.01 (m, 3 H), 2.95 (dd, *J* = 4.8 Hz, 1 H), 2.64 (brm, 1 H), 2.55-2.39 (m, 2 H), 2.24-2.23 (m, 1 H), 1.97-1.96 (m, 2 H), 1.74-1.70 (m, 1 H), 1.58-1.53 (m, 8 H), 1.47-1.43 (m, 3 H), 1.36-1.34 (m, 3 H), 1.26-1.14 (m, 6 H); ^{13}C NMR ($CDCl_3$, 100 MHz) δ = 211.2, 173.0, 172.2, 171.8, 170.8, 137.5, 136.2, 129.4, 129.3, 128.6, 128.5, 127.2, 126.7, 111.6, 79.9, 66.1, 60.8, 56.8, 54.2, 53.8, 46.9, 38.5, 37.6, 36.6, 35.7, 34.5, 30.3, 29.7, 28.7, 27.9, 25.7, 25.0, 24.5, 23.9, 23.7; HRMS (QTOF ES+) found *m/z* 709.3561 $(M + Na)^+$, $C_{39}H_{50}N_4NaO_7$ requires 709.3577.

*(3S,6S,9S,14aR)-6,9-Dibenzyl-3-((R)-7,8-dihydroxy-6-oxooctyl)decahydropyrrolo[1,2-a][1,4,7,10]tetraazacyclododecine-1,4,7,10-tetraone (**25**).*

Aqueous HCl (1.5 N, 2 mL) was added drop wise to a stirred solution of compound **24** (100 mg, 0.15 mmol) in THF (2 mL) at 0 ℃ and stirring was continued for 5 h before being diluted with water (50 mL) and neutralized with solid $NaHCO_3$ (1.5 g). It was then extracted with dichloromethane (2 × 25 mL) and the combined organic extract was washed successively with water (25 mL), brine (25 mL), and then dried over $MgSO_4$. It was then filtered and the filtrate was concentrated in vacuo to leave a crude which was purified by chromatography over silica gel using ethyl acetate- hexane mixture (1:1) to provide **25** as a colorless foam (64 mg, 71%): $[\alpha]_D^{25}$ +66.5 (*c* 1.8, $CHCl_3$); IR ($CHCl_3$) 3354, 2935, 2864, 1755, 1711, 1516, 1420, 1367, 1165 cm^{-1}; ^1H NMR ($CDCl_3$, 400 MHz) δ = 7.52 (brs, 1 H), 7.26-6.99 (m, 10 H), 4.66 (brd, 1 H), 4.39-4.36 (m, 1 H), 4.19-

4.09 (m, 2 H), 3.94 (s, 1 H), 3.20-3.06 (m, 3 H), 2.67-2.25 (m, 1 H), 1.94 (s, 1 H), 1.67-1.43 (m, 16 H), 1.28-1.21 (m, 5 H); ^{1}H NMR (DMSO-d_6, 400 MHz) δ = 8.32 (s, 1 H), 7.82 (brs, 1 H), 7.36-7.18 (m, 11 H), 5.16 (d, *J* = 5.6 Hz, 1 H),4.75 (t, *J* = 5.6 Hz, 1 H), 4.62 (t, *J* = 7.2, 1H), 4.48 (t, *J* = 6.4 Hz, 1 H), 4.34-4.20 (m, 4 H), 4.10 (t, *J* = 8.0 Hz, 1 H), 3.92 (q, *J* = 4.8 Hz, 1 H), 3.86 (dd, *J* = 5.2, 8.4 Hz, 1 H), 3.48-3.47 (m, 1 H), 2.92 (d, *J* = 12.4 Hz, 3 H), 1.97 (brm, 2 H), 1.76-1.68 (m, 4 H), 1.55-1.53 (m, 4 H), 1.36-1.32 (m, 4 H). ^{13}C NMR ($CDCl_3$, 100 MHz) δ = 210.7, 173.1, 172.1, 171.4, 171.0, 137.5, 136.0, 129.4, 129.3, 128.6, 128.5, 127.3, 126.7, 79.9, 63.7, 60.7, 56.6, 53.9, 53.8, 46.9, 38.4, 37.4, 35.6, 31.6, 29.7, 28.0, 27.9, 25.0, 24.4; HRMS (QTOF ES+) found *m/z* 629.2949 $(M + Na)^+$, $C_{33}H_{42}N_4NaO_7$ requires 629.2951.

SS-1-55 16.01.2015

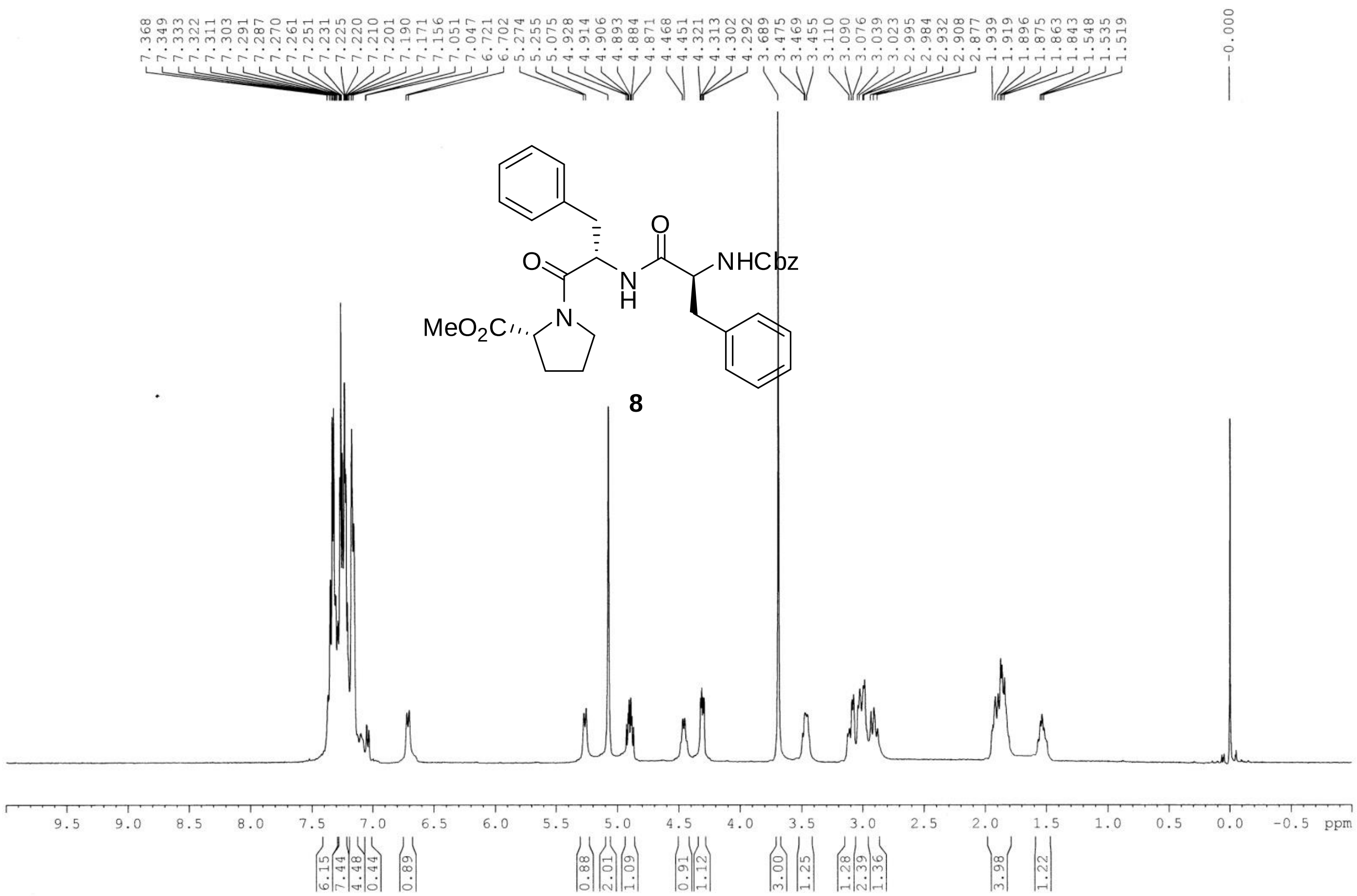

^{1}H NMR spectrum of compound **8** in $CDCl_3$.

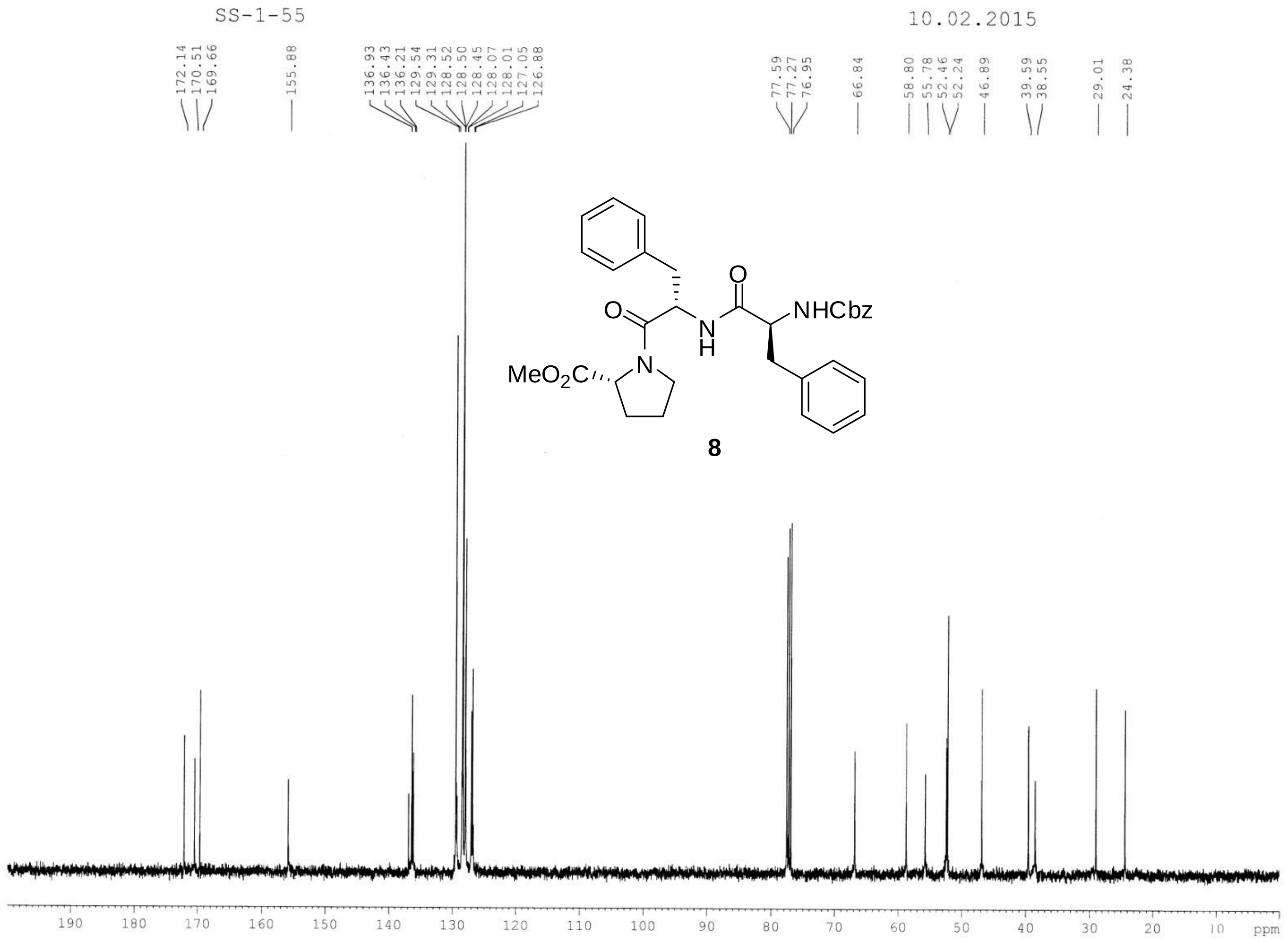

^{13}C NMR spectrum of compound **8** in $CDCl_3$.

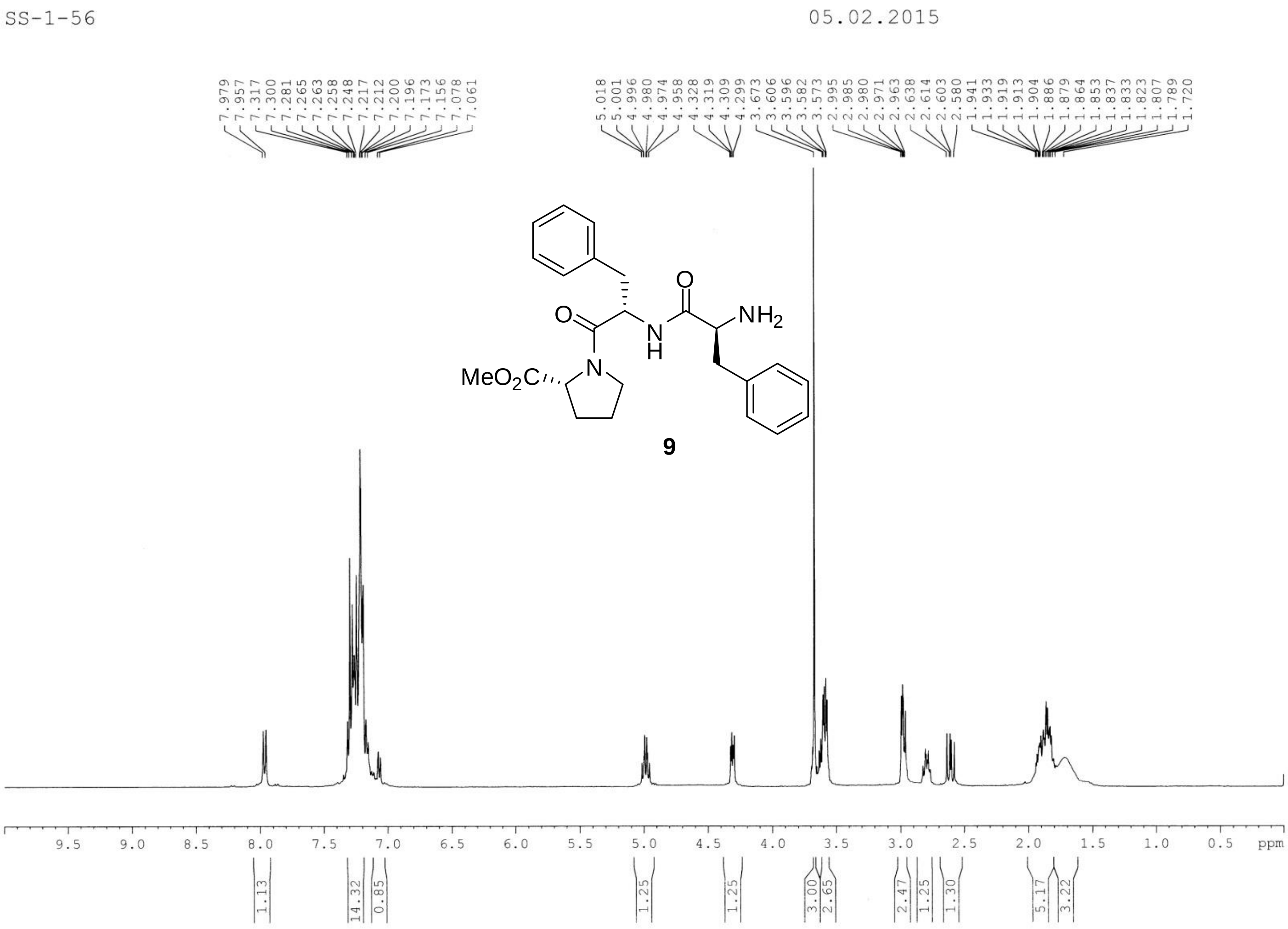

^{1}H NMR spectrum of compound **9** in $CDCl_3$.

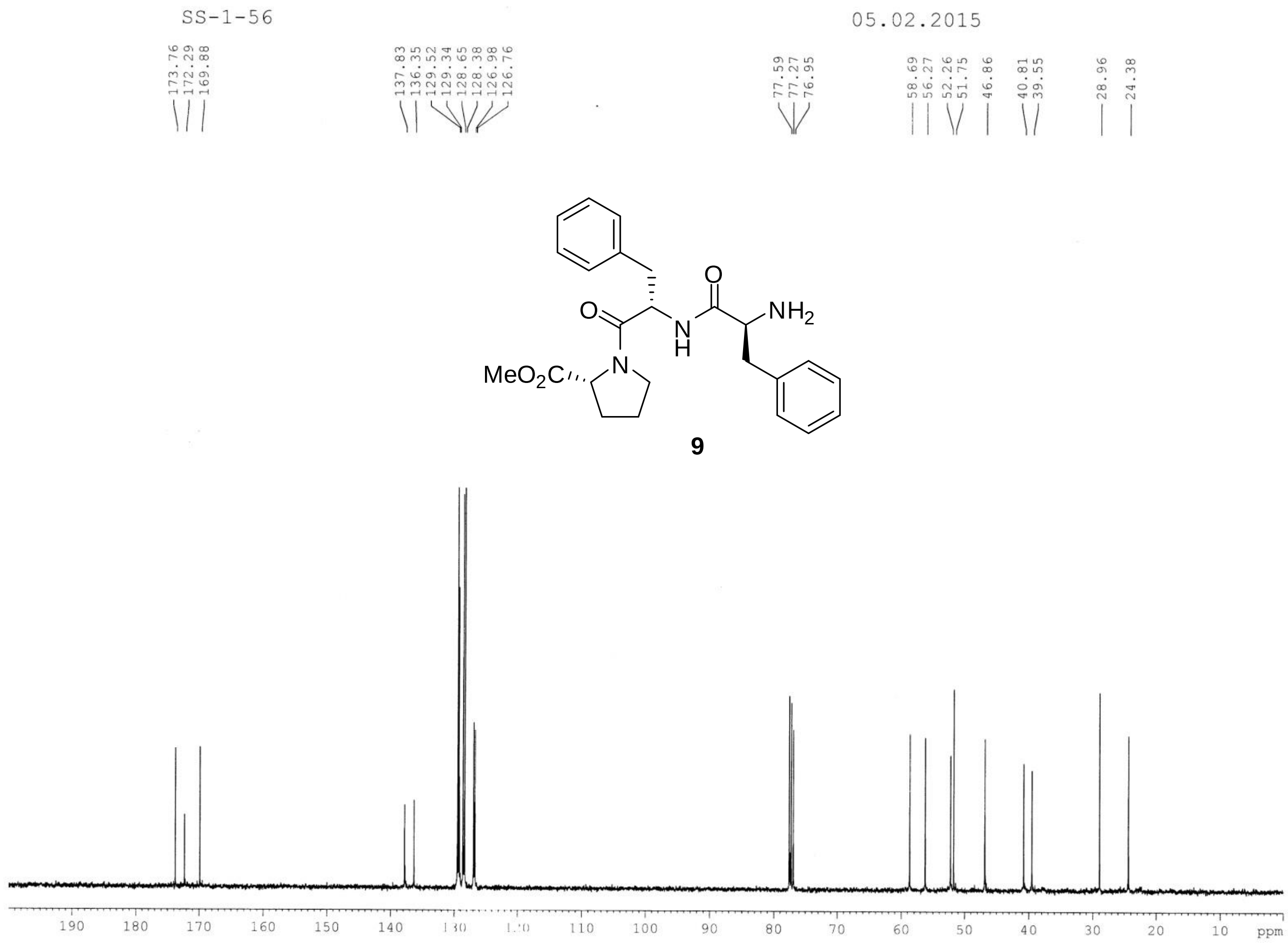

^{13}C NMR spectrum of compound **9** in $CDCl_3$.

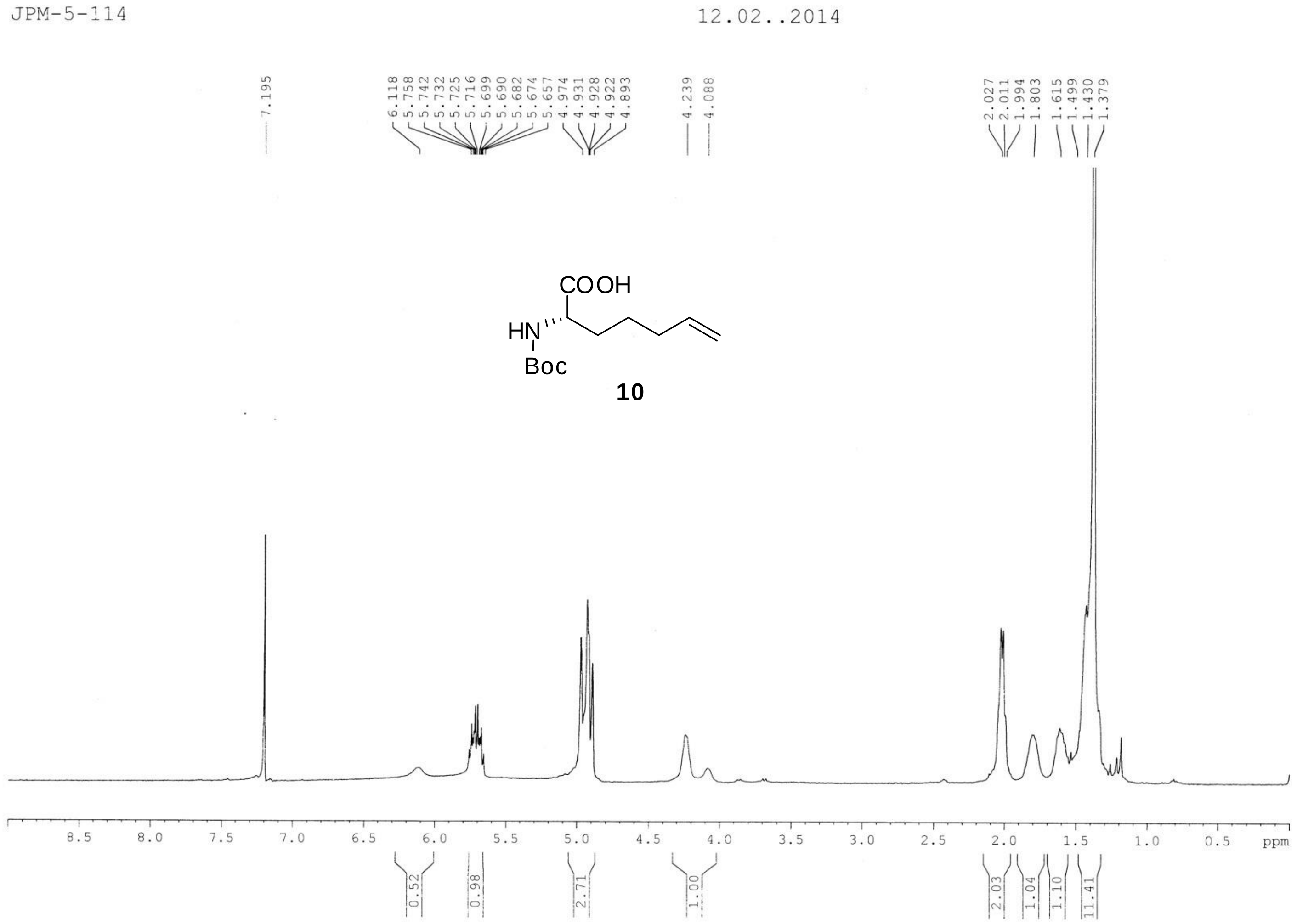

^{1}H NMR spectrum of compound **10** in $CDCl_3$.

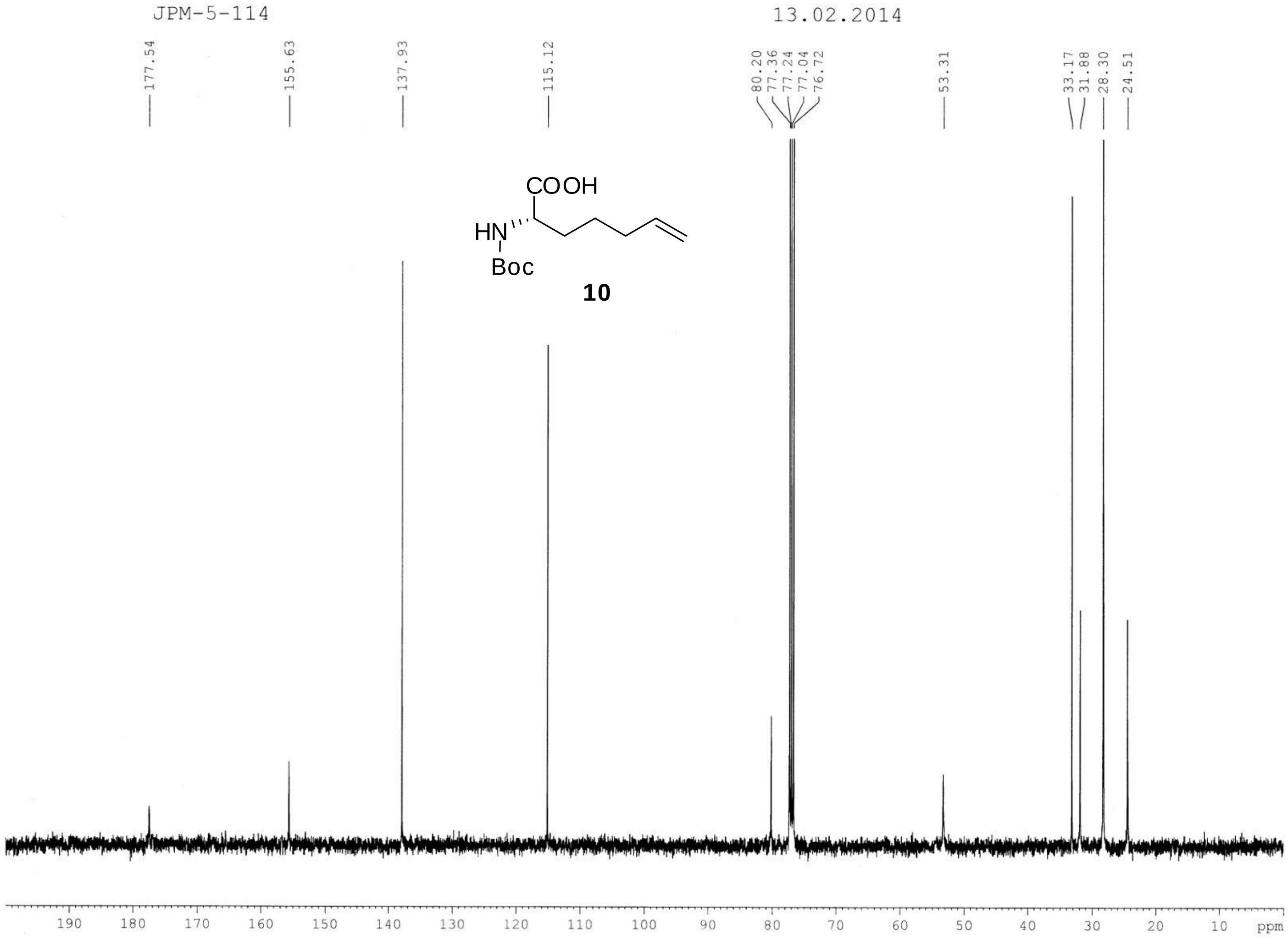

^{13}C NMR spectrum of compound **10** in $CDCl_3$.

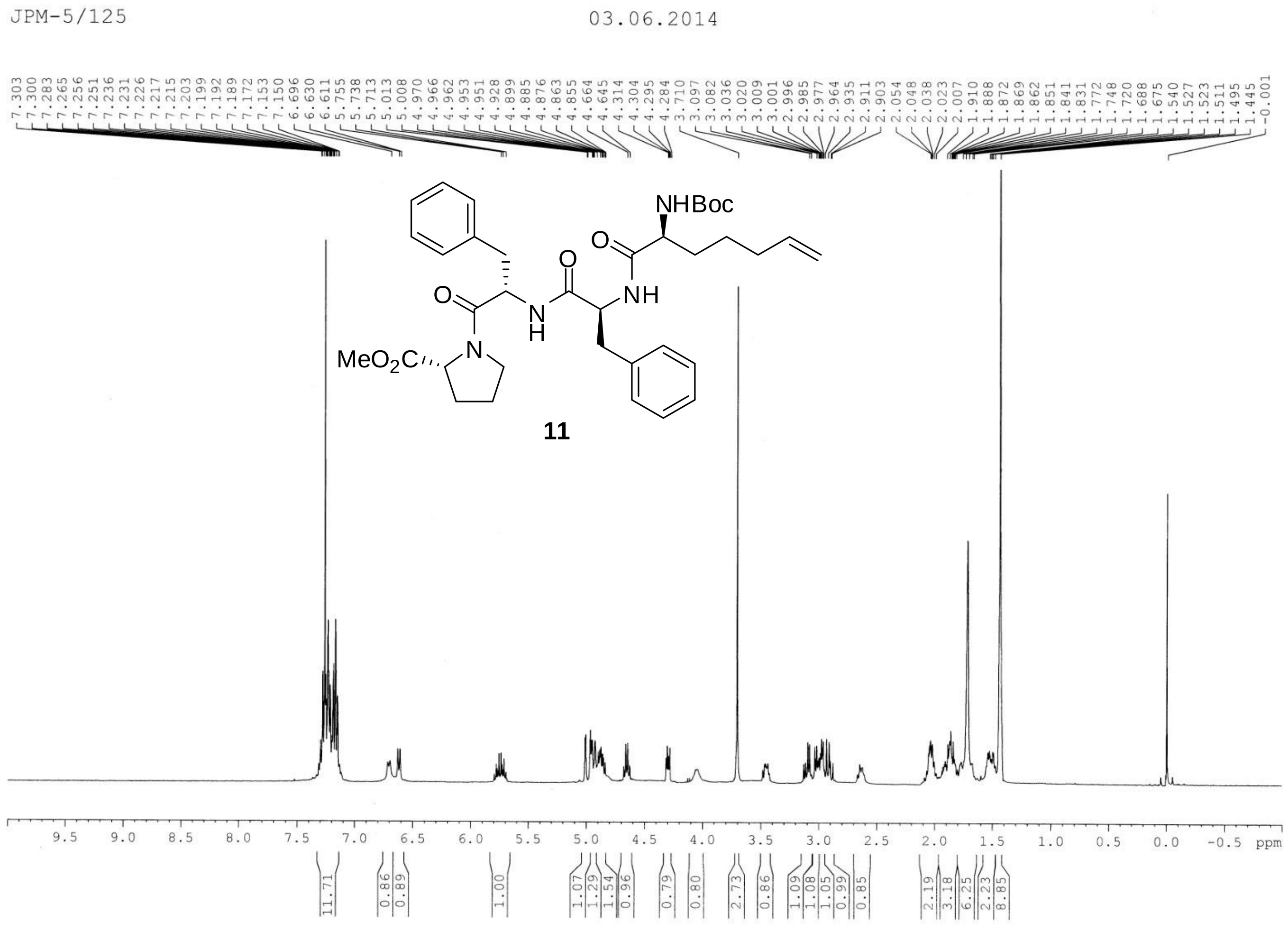

^{1}H NMR spectrum of compound **11** in $CDCl_3$.

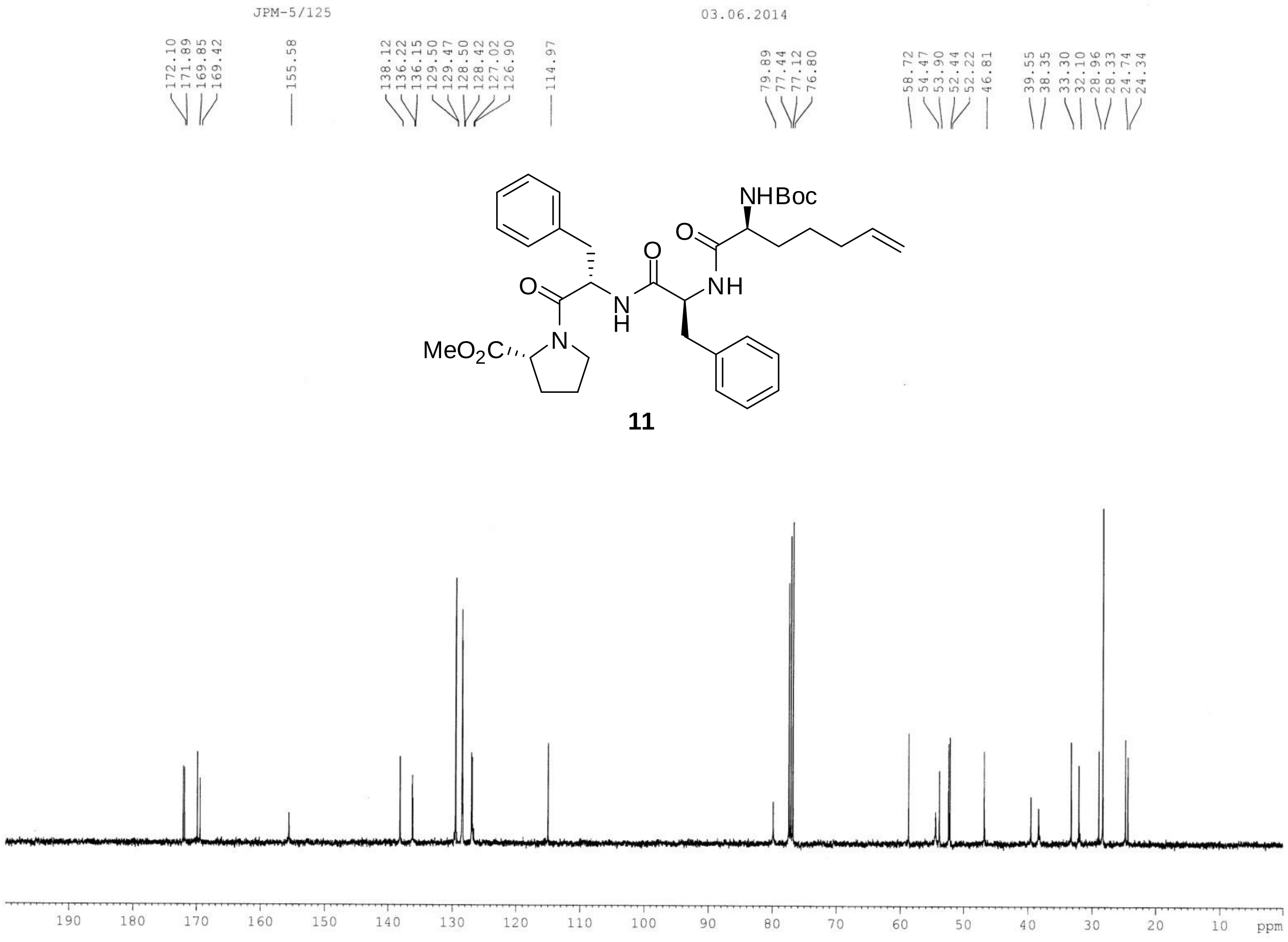

^{13}C NMR spectrum of compound **11** in $CDCl_3$.

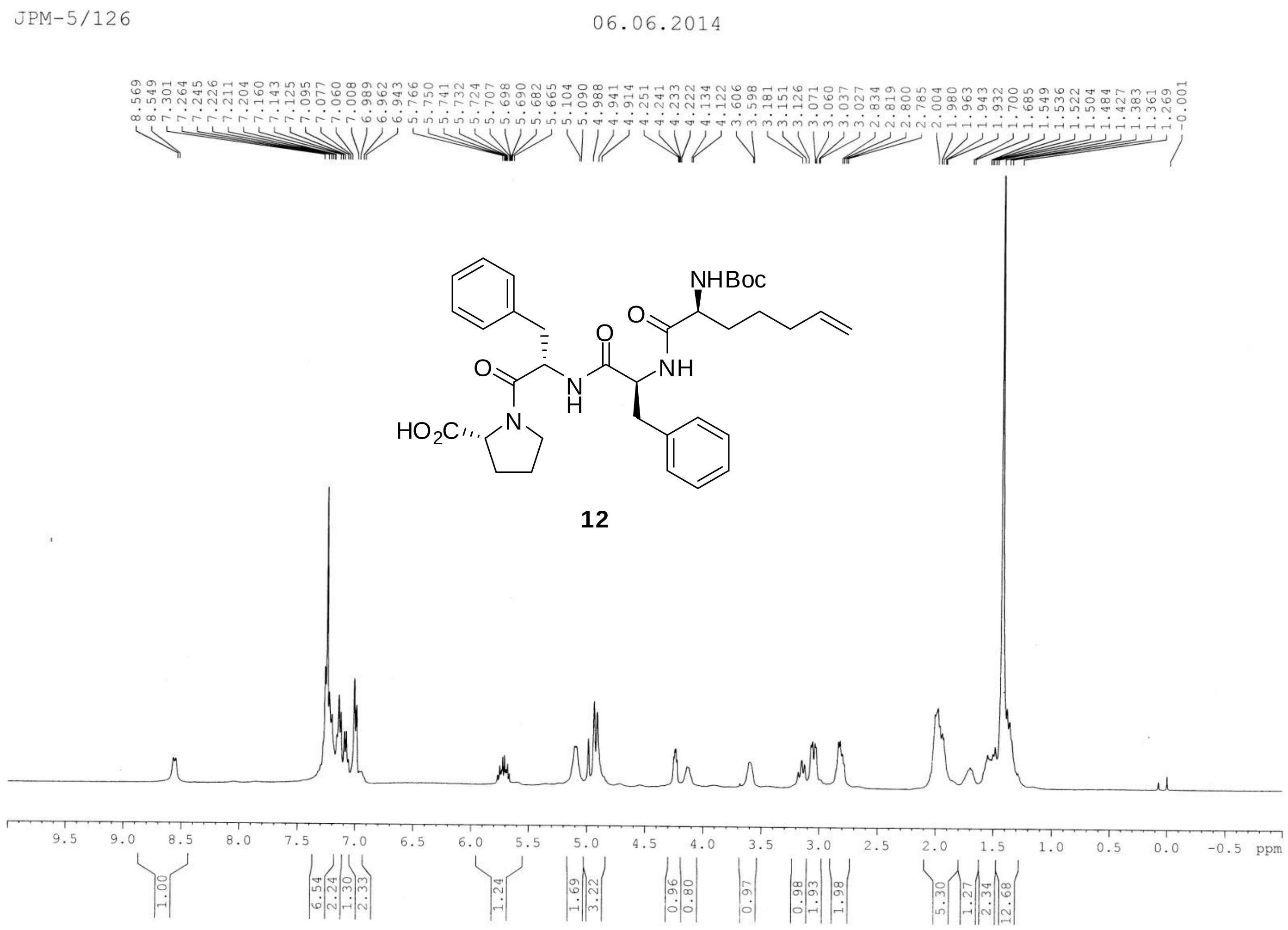

^{1}H NMR spectrum of compound **12** in $CDCl_3$.

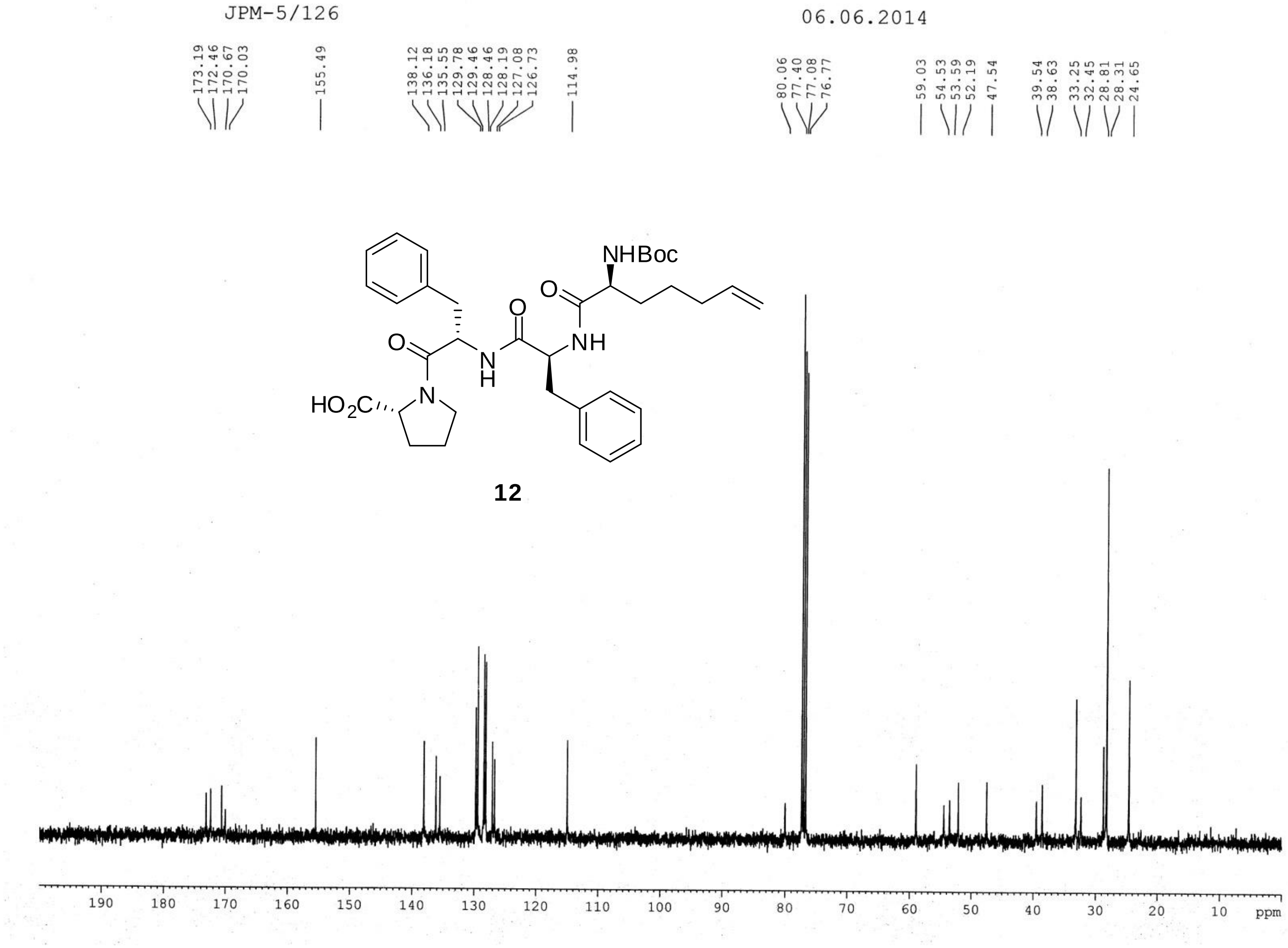

^{13}C NMR spectrum of compound **12** in $CDCl_3$.

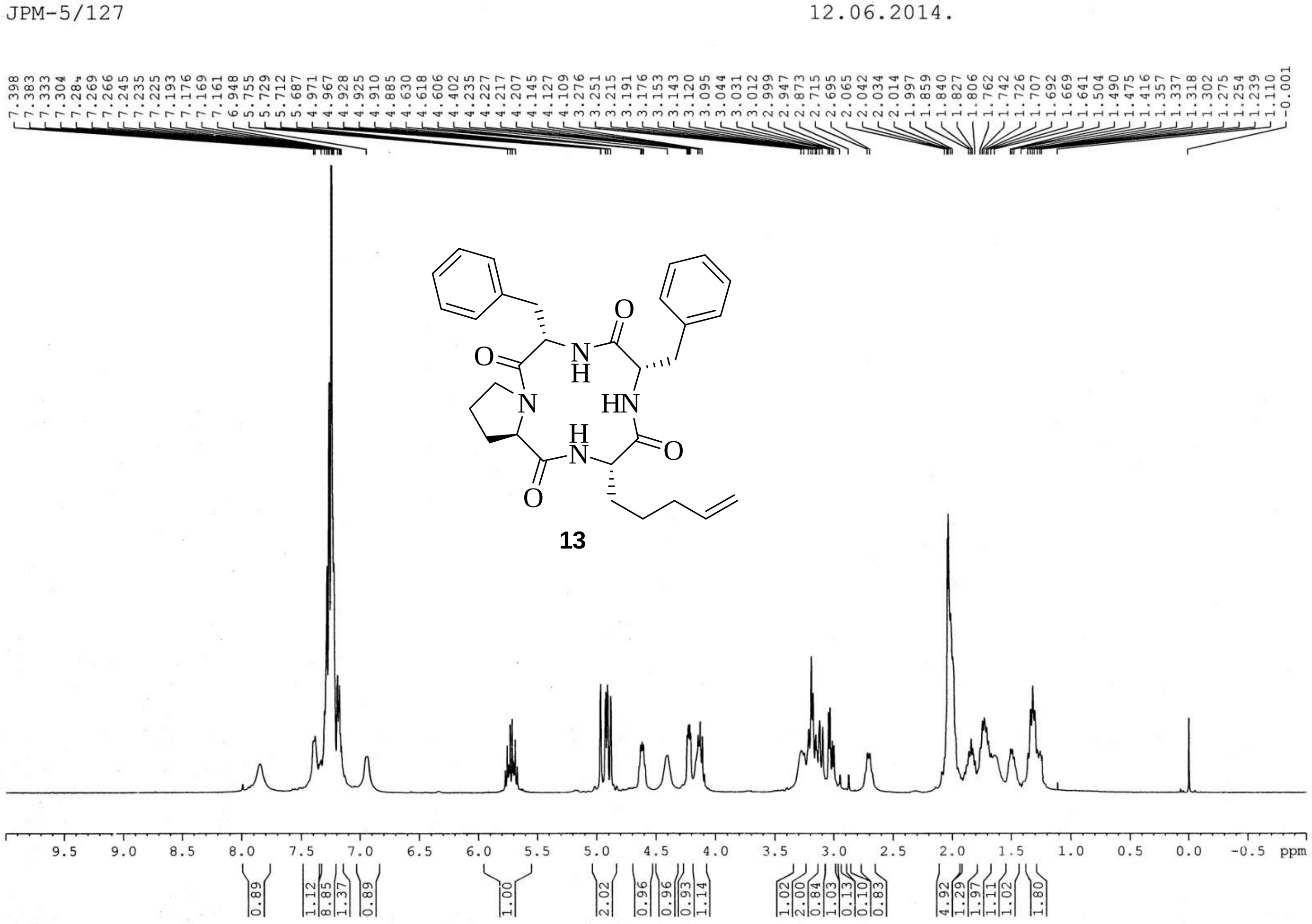

^{1}H NMR spectrum of compound **13** in $CDCl_3$.

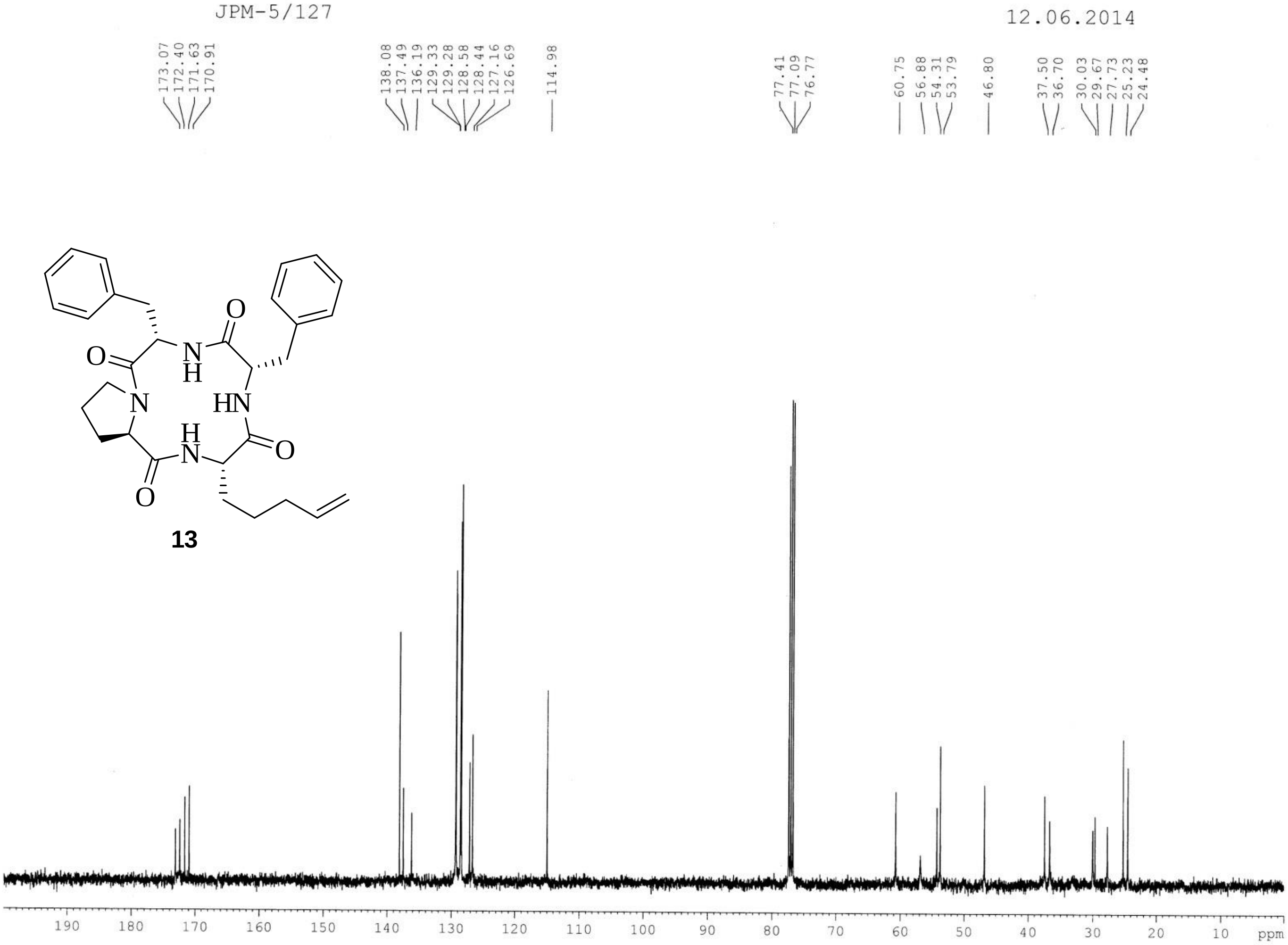

^{13}C NMR spectrum of compound **13** in $CDCl_3$.

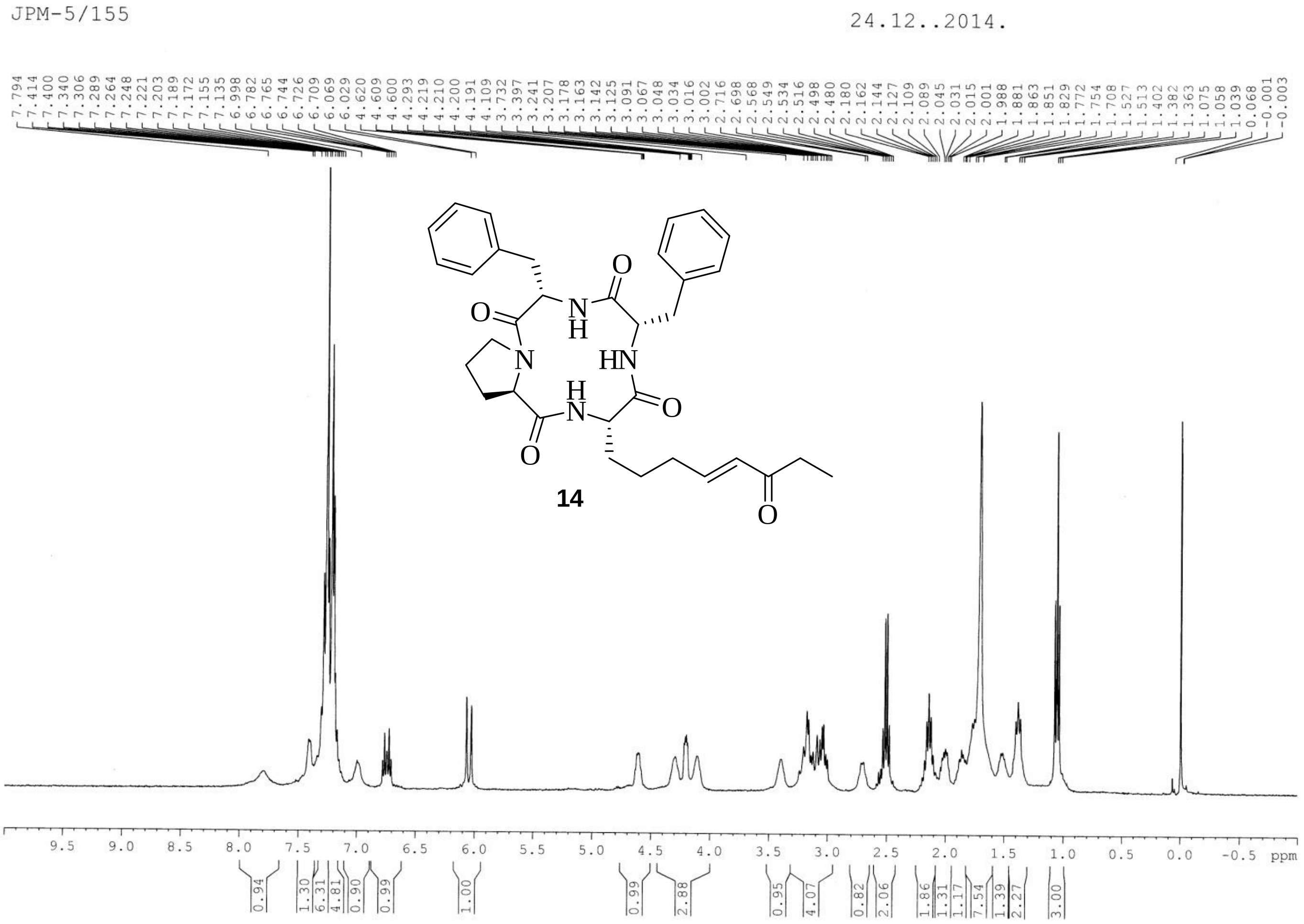

^{1}H NMR spectrum of compound **14** in $CDCl_3$.

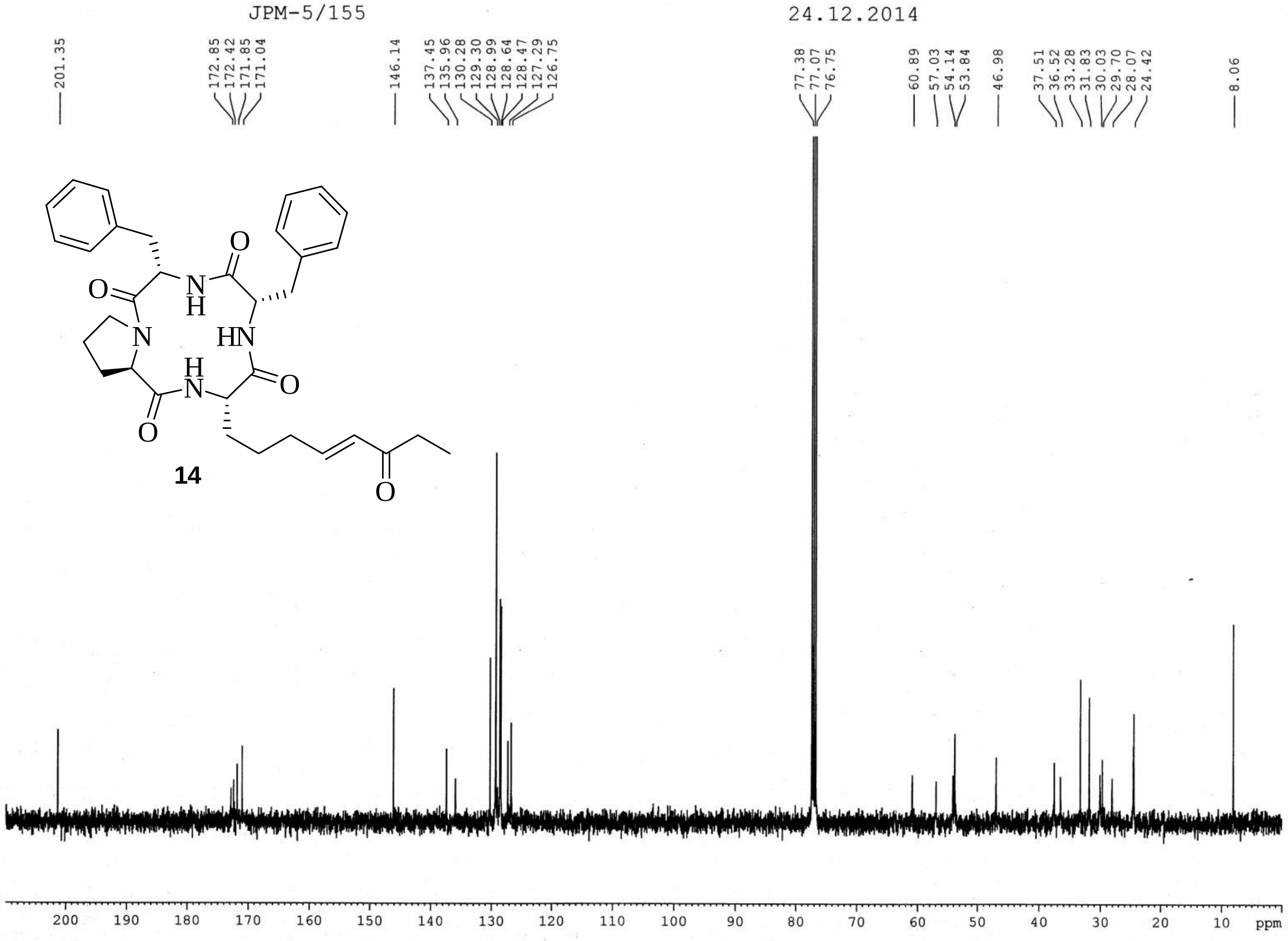

^{13}C NMR spectrum of compound **14** in $CDCl_3$.

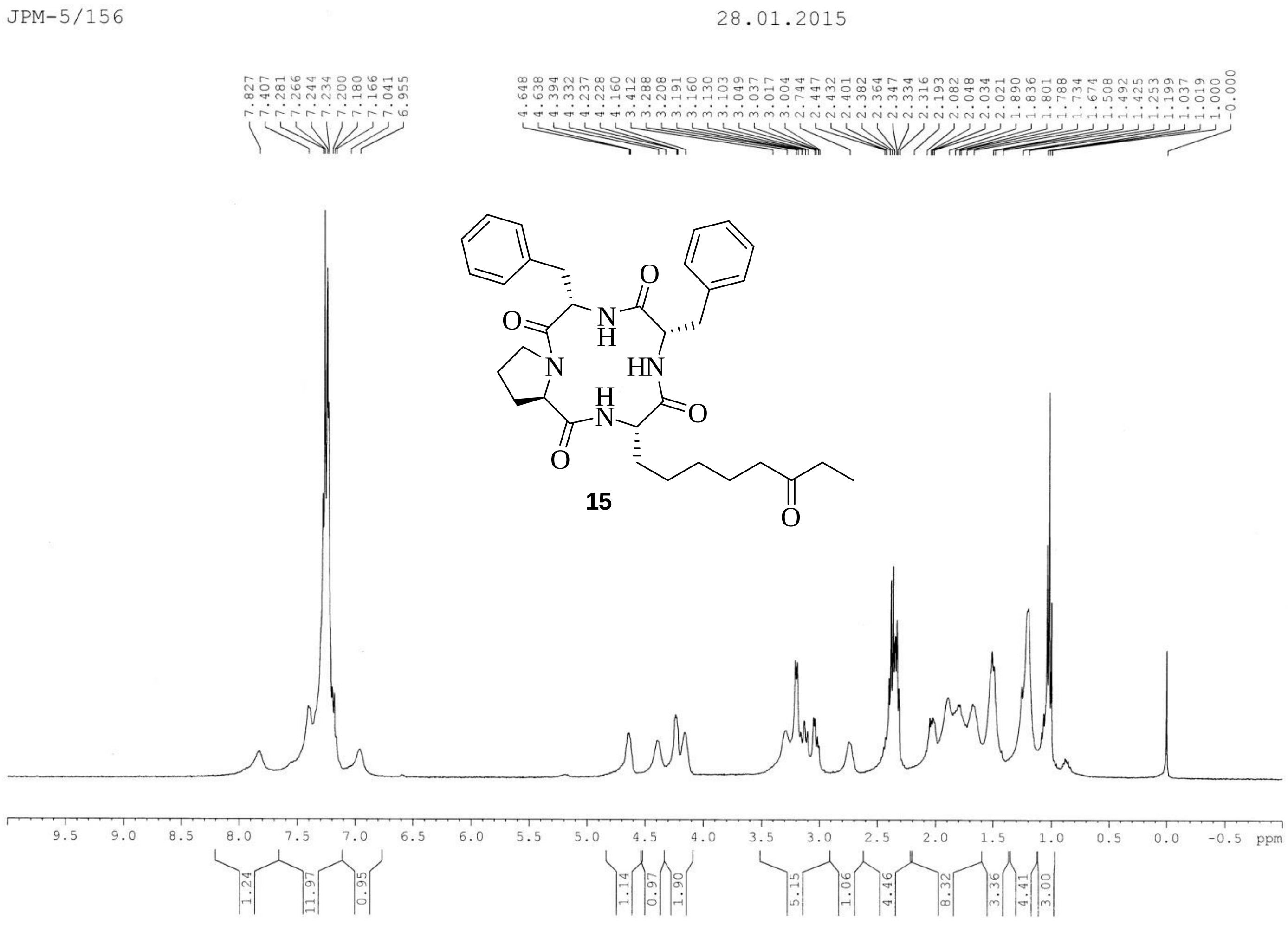

^{1}H NMR spectrum of compound **15** in $CDCl_3$.

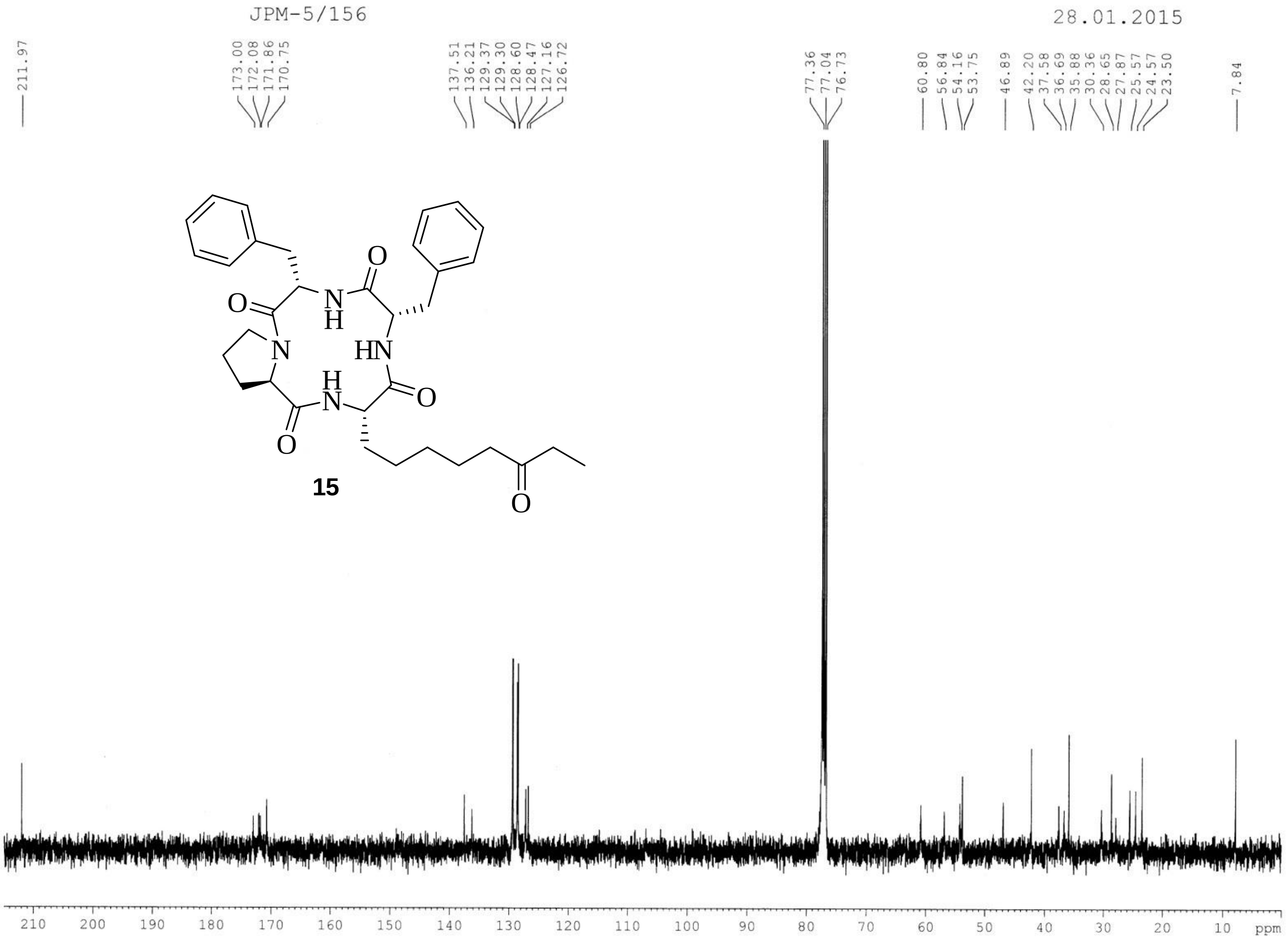

^{13}C NMR spectrum of compound **15** in $CDCl_3$.

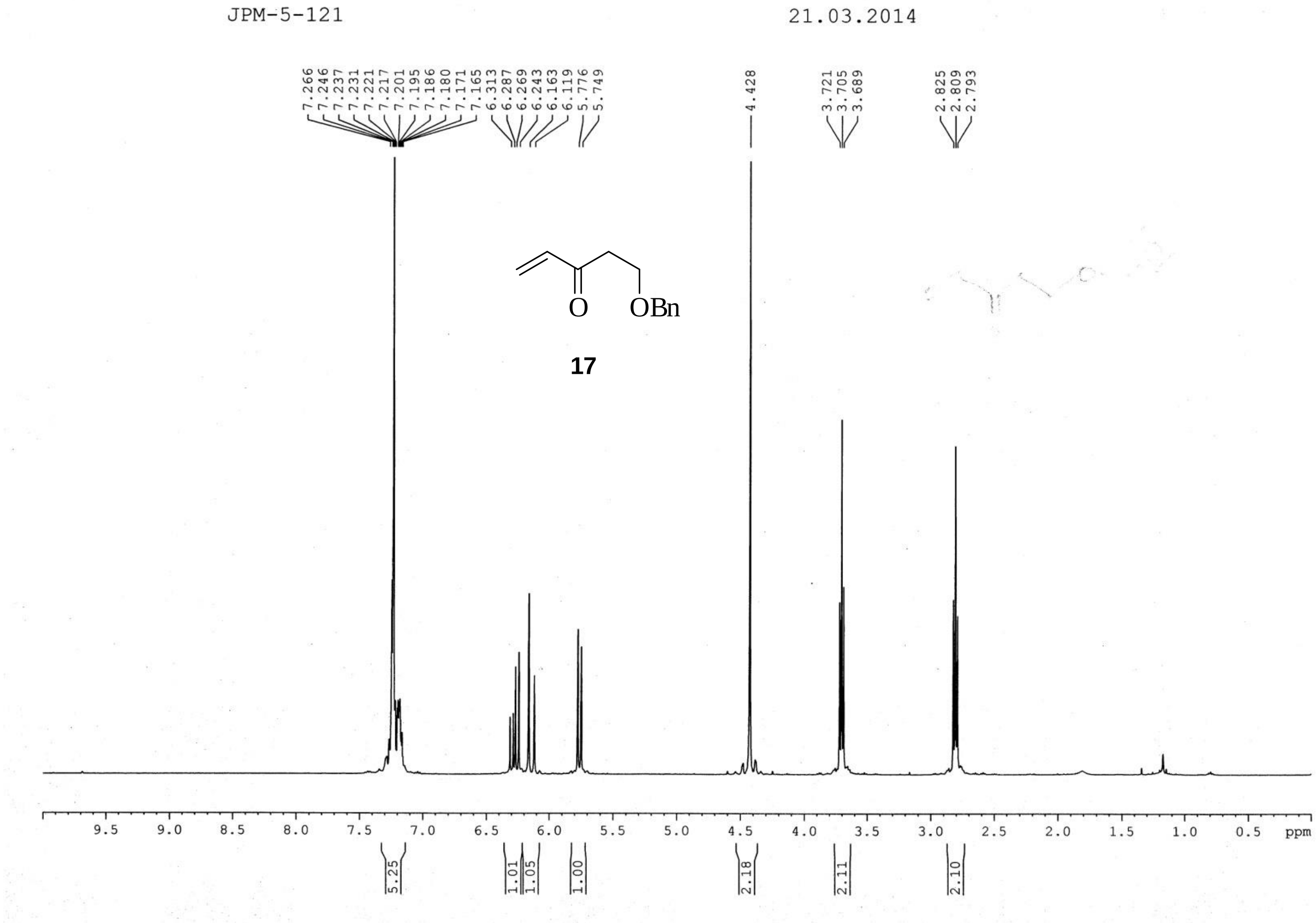

^{1}H NMR spectrum of compound **17** in $CDCl_3$.

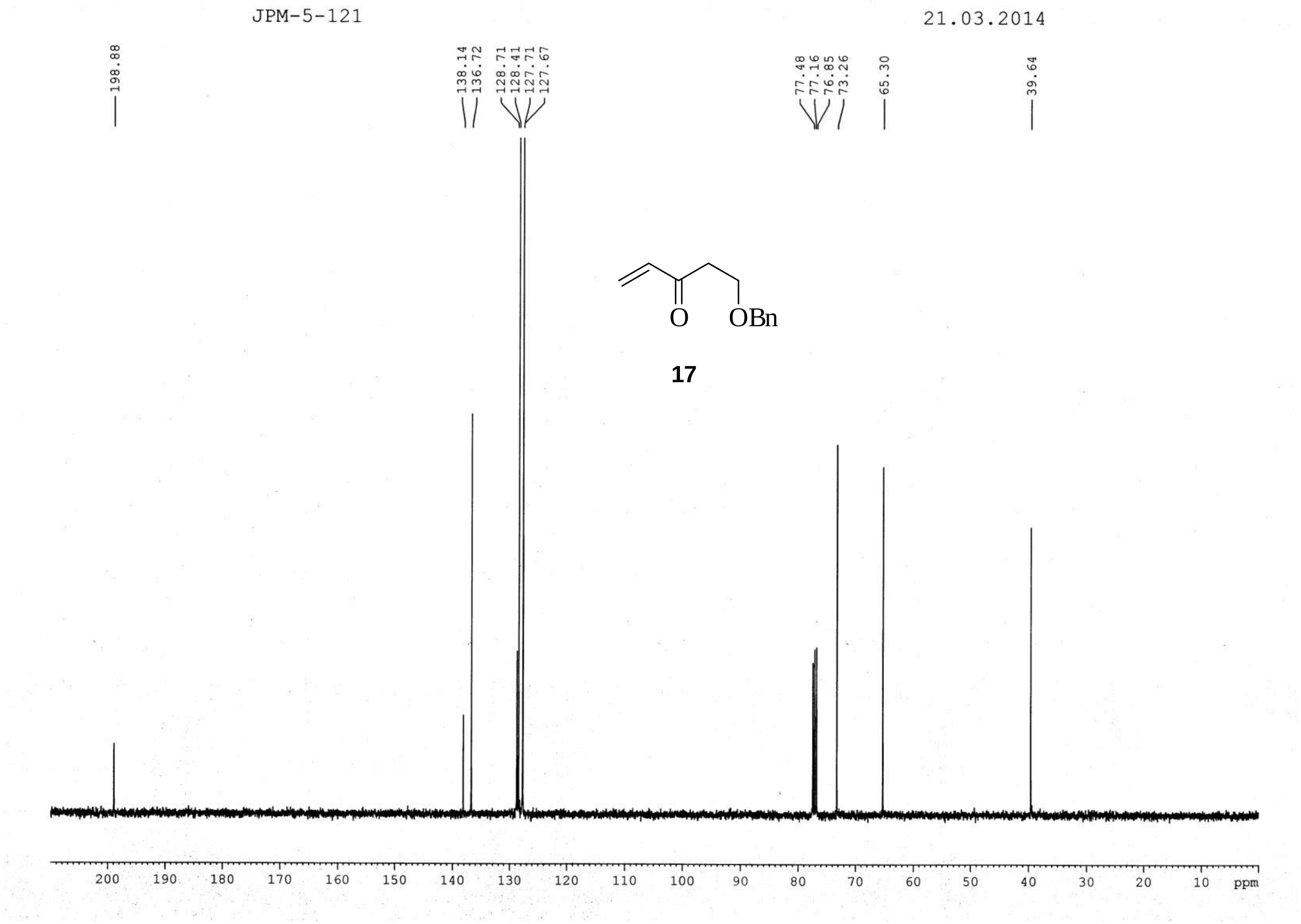

^{13}C NMR spectrum of compound **17** in $CDCl_3$.

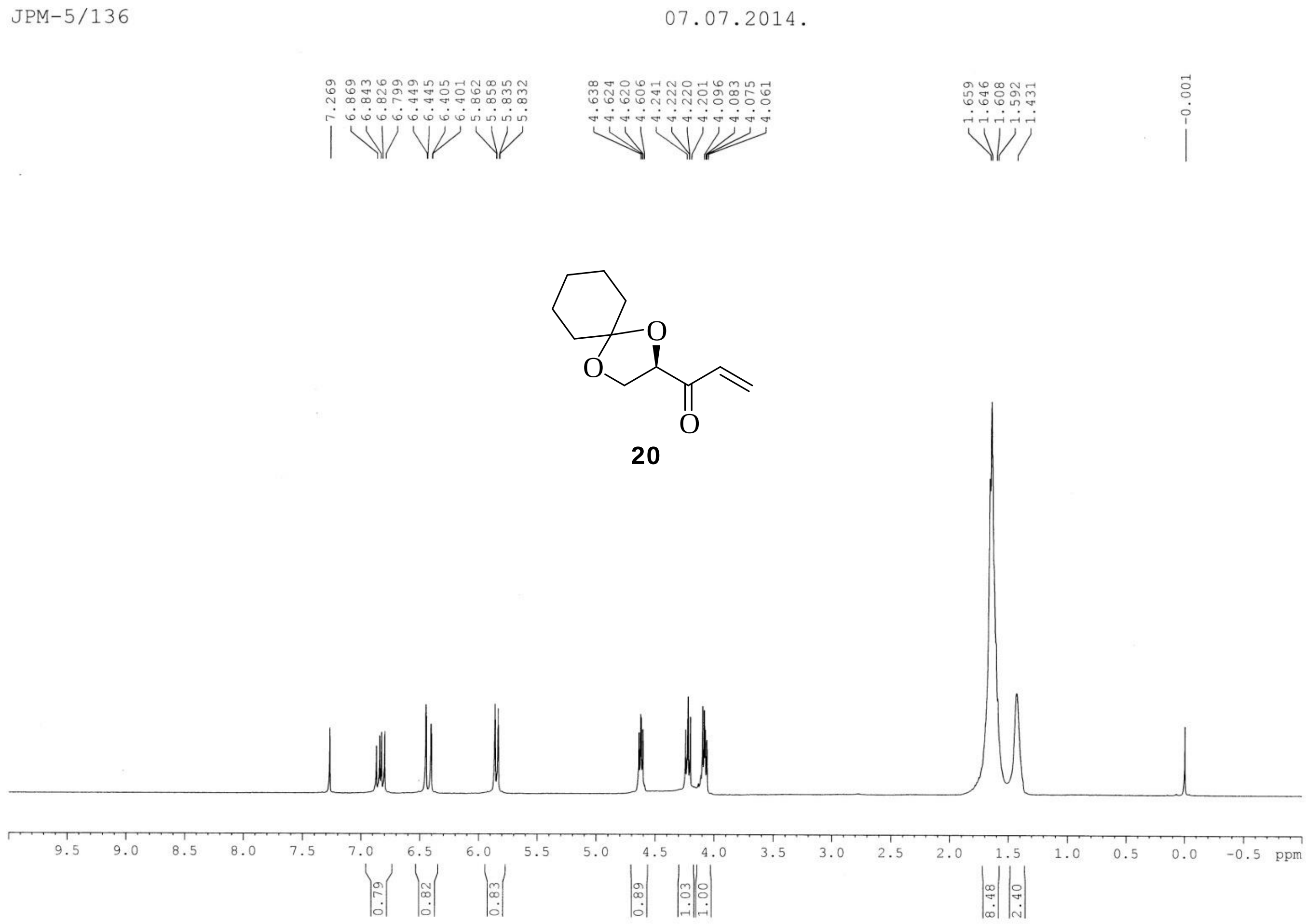

^{1}H NMR spectrum of compound **20** in $CDCl_3$.

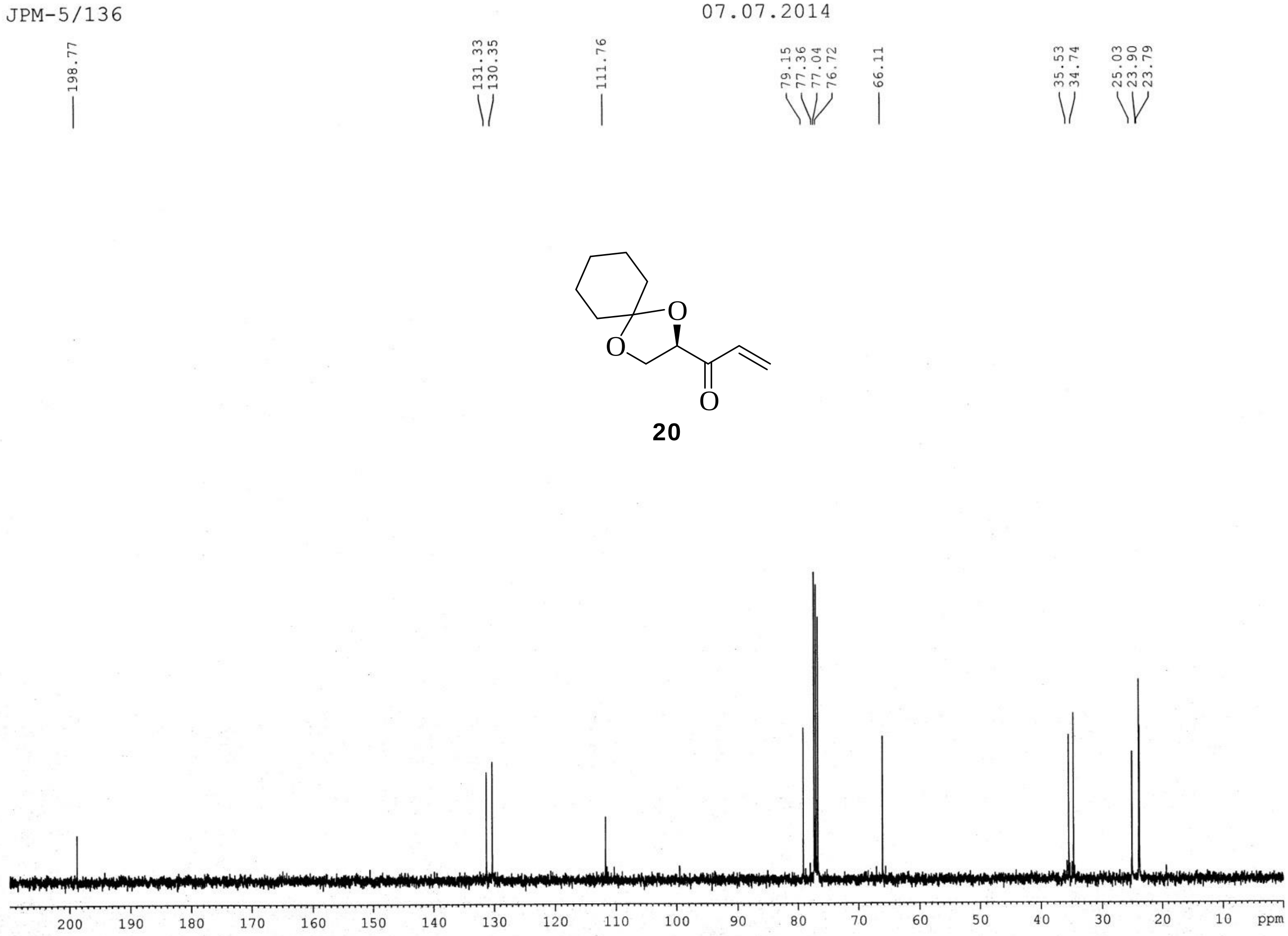

^{13}C NMR spectrum of compound **20** in $CDCl_3$.

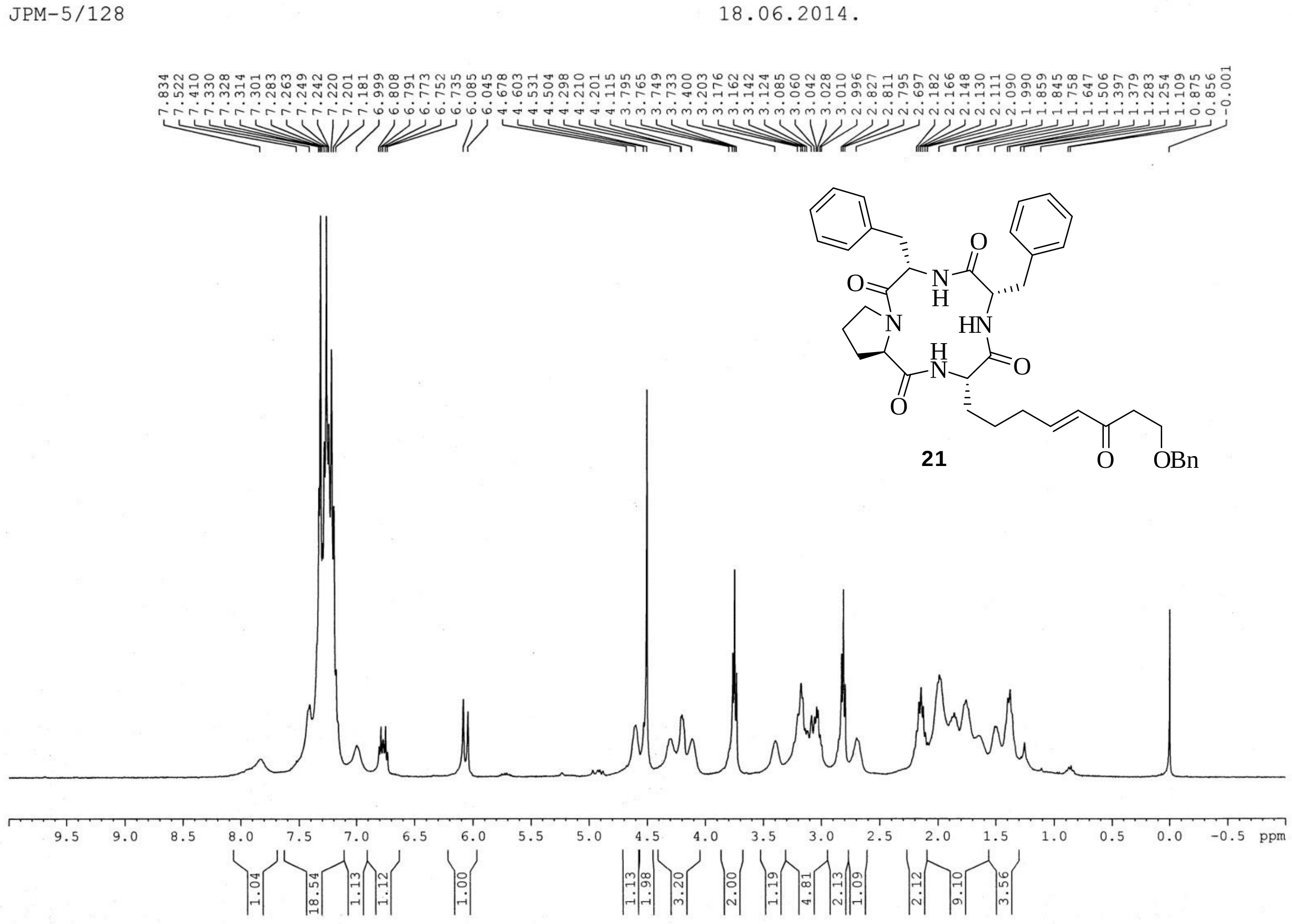

^{1}H NMR spectrum of compound **21** in $CDCl_3$.

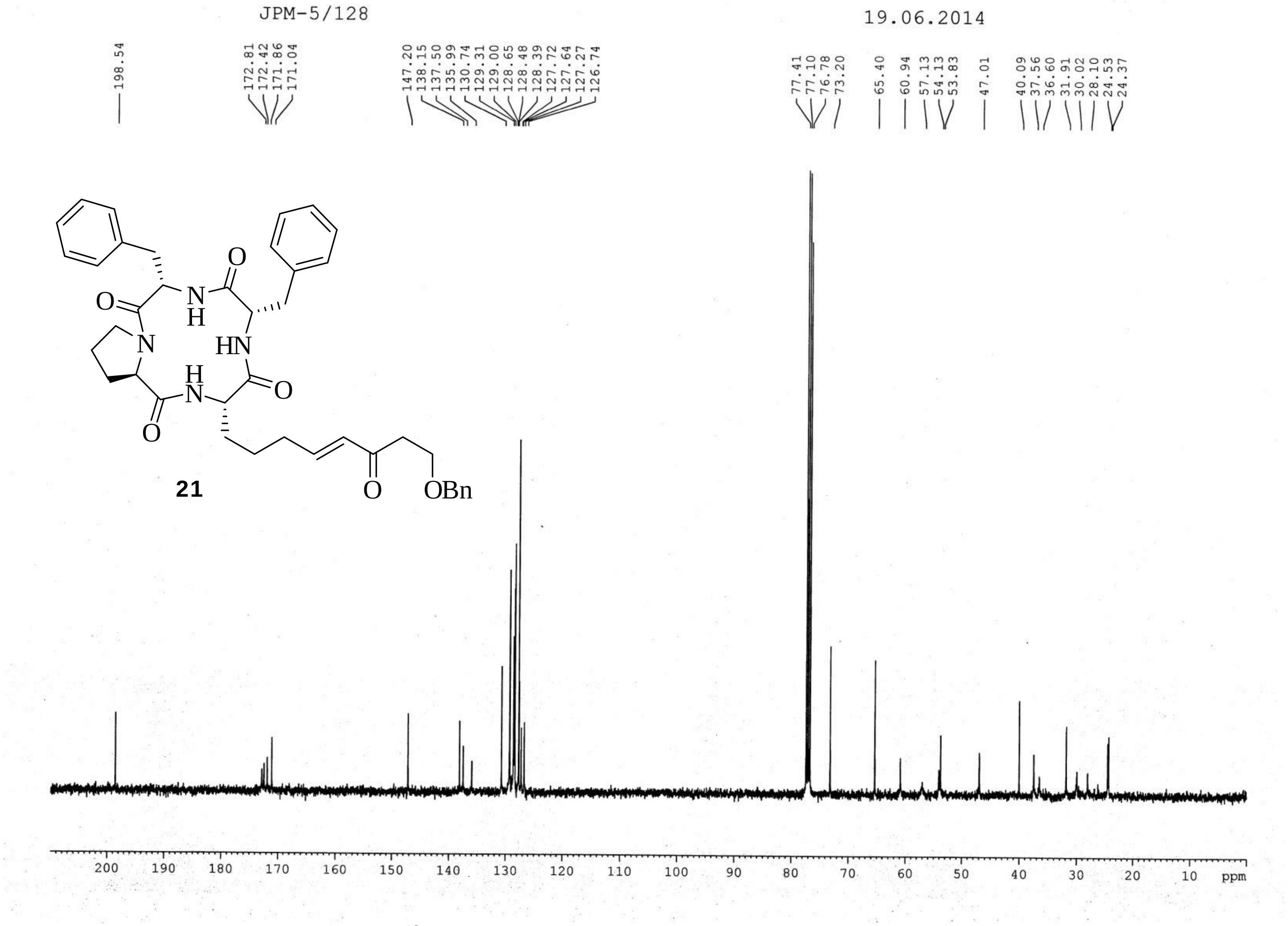

^{13}C NMR spectrum of compound **21** in $CDCl_3$.

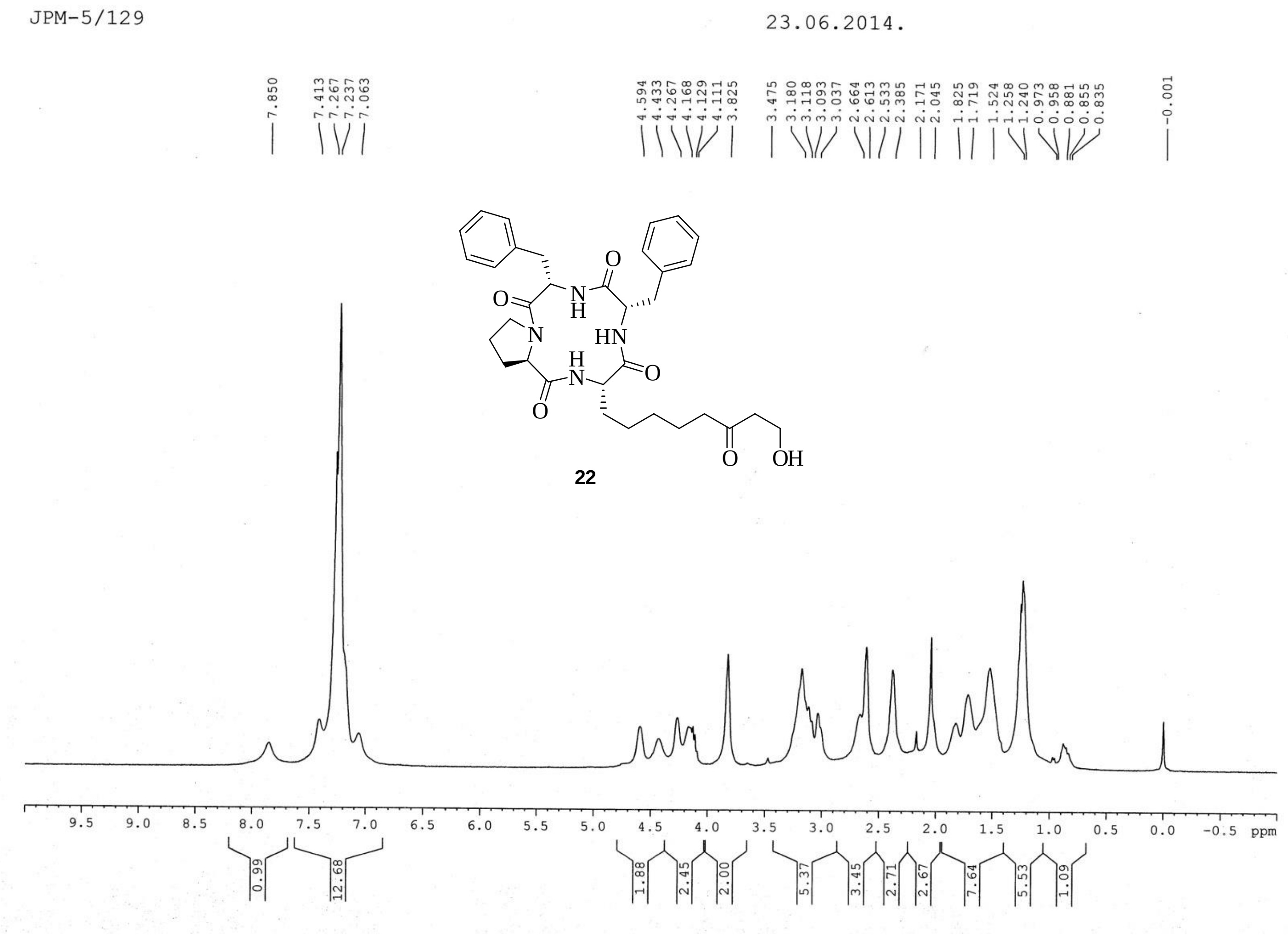

^1H NMR spectrum of compound **22** in $CDCl_3$.

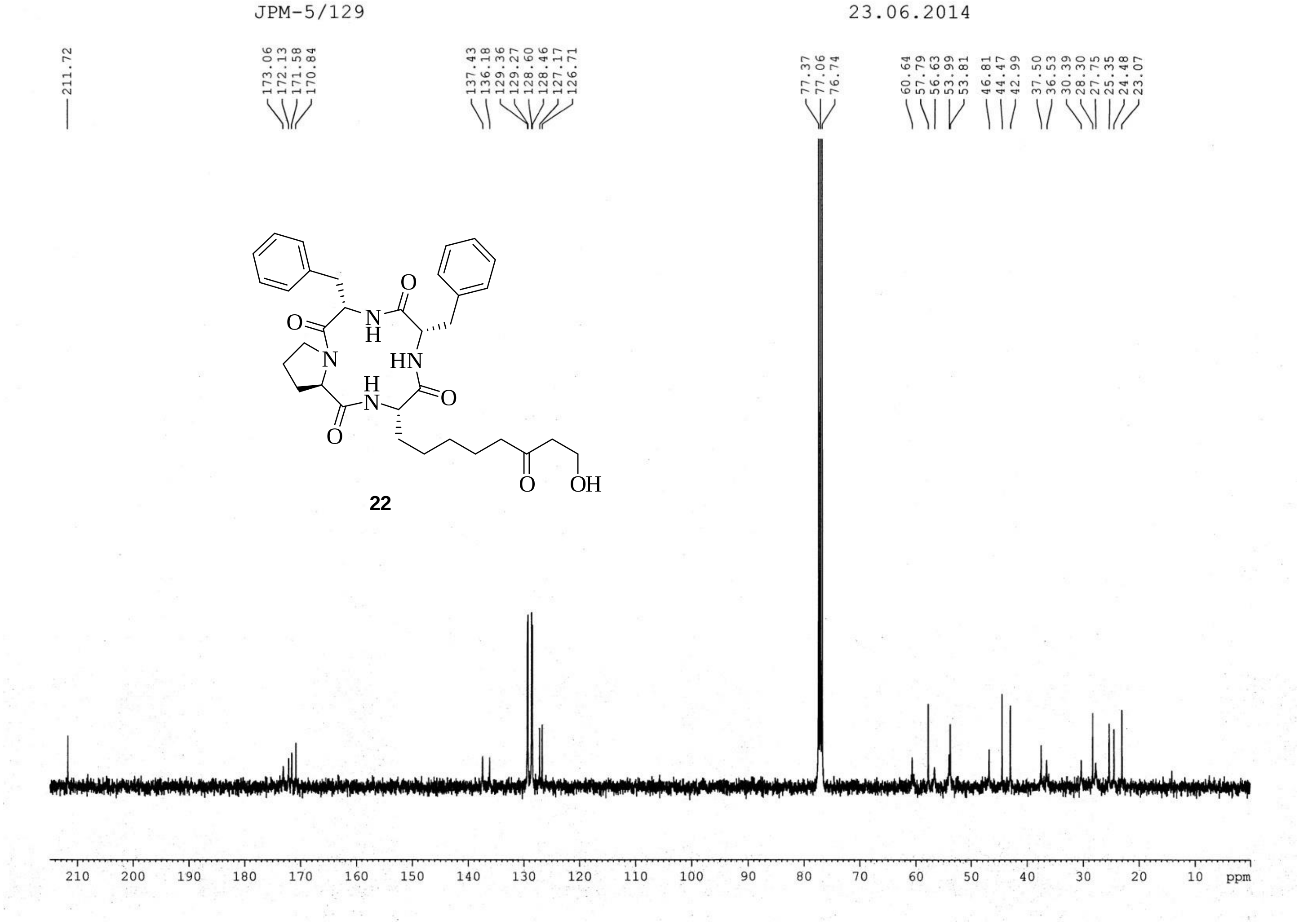

^{13}C NMR spectrum of compound **22** in $CDCl_3$.

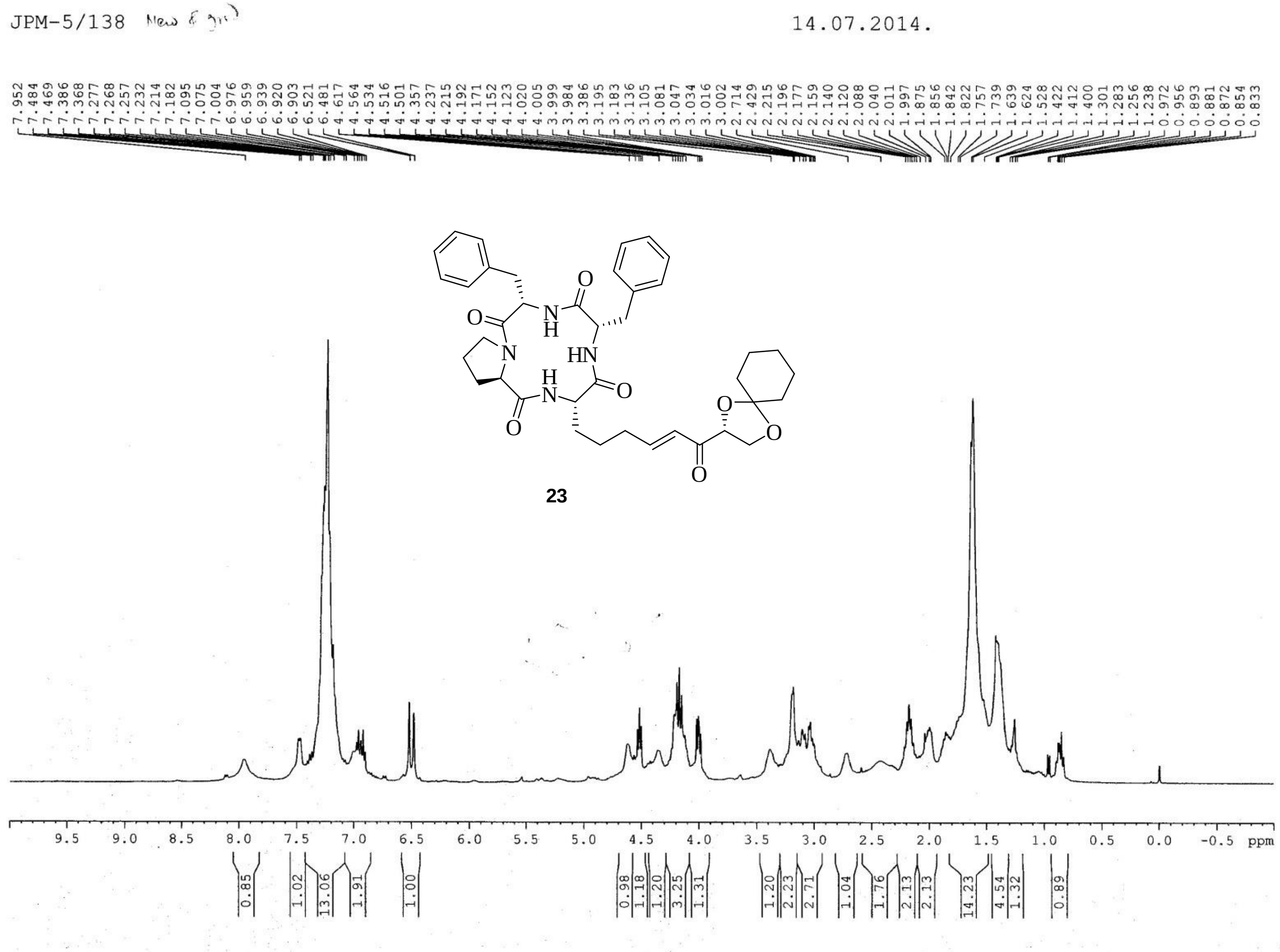

^{1}H NMR spectrum of compound **23** in $CDCl_3$.

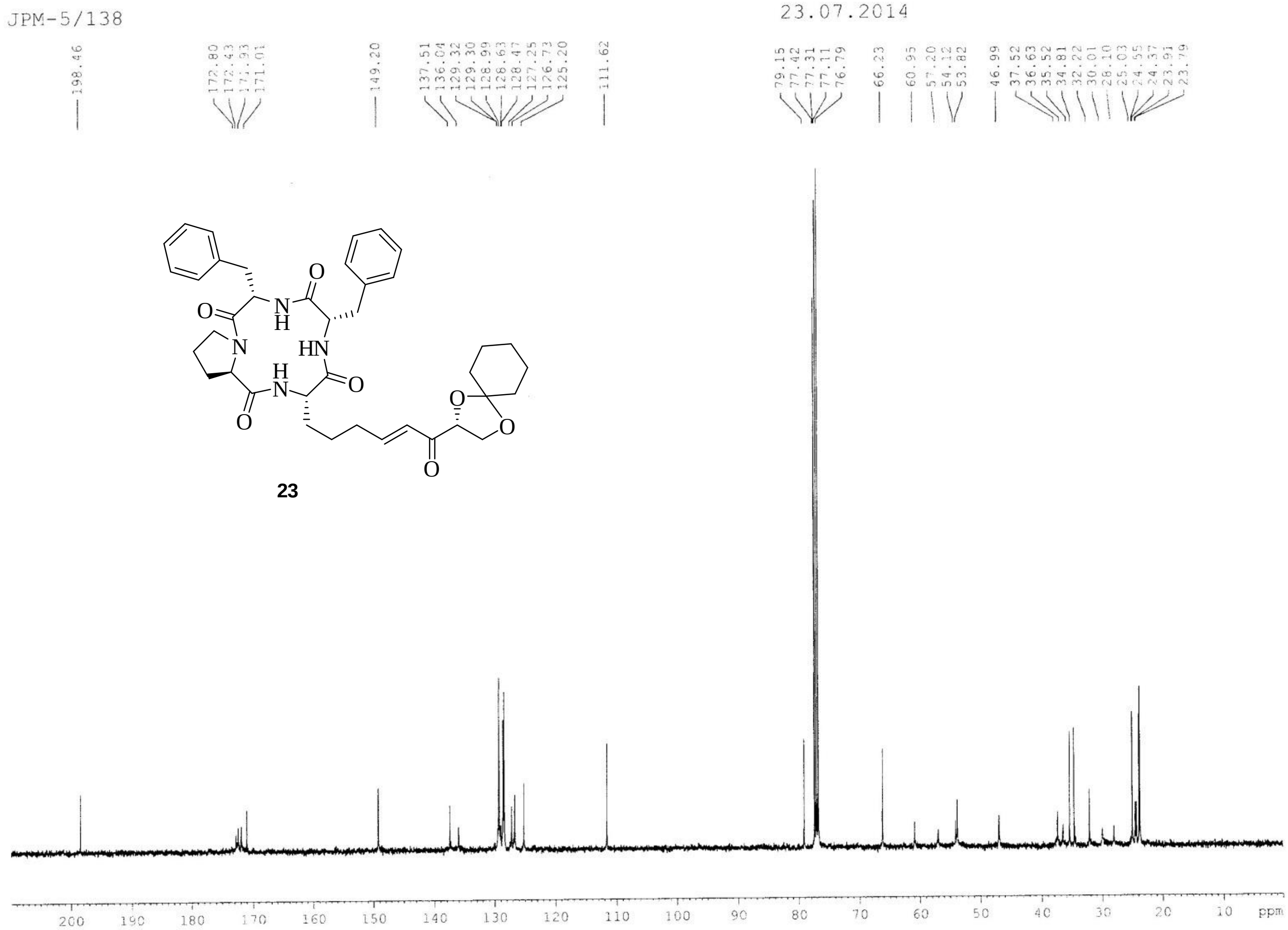

^{13}C NMR spectrum of compound **23** in $CDCl_3$.

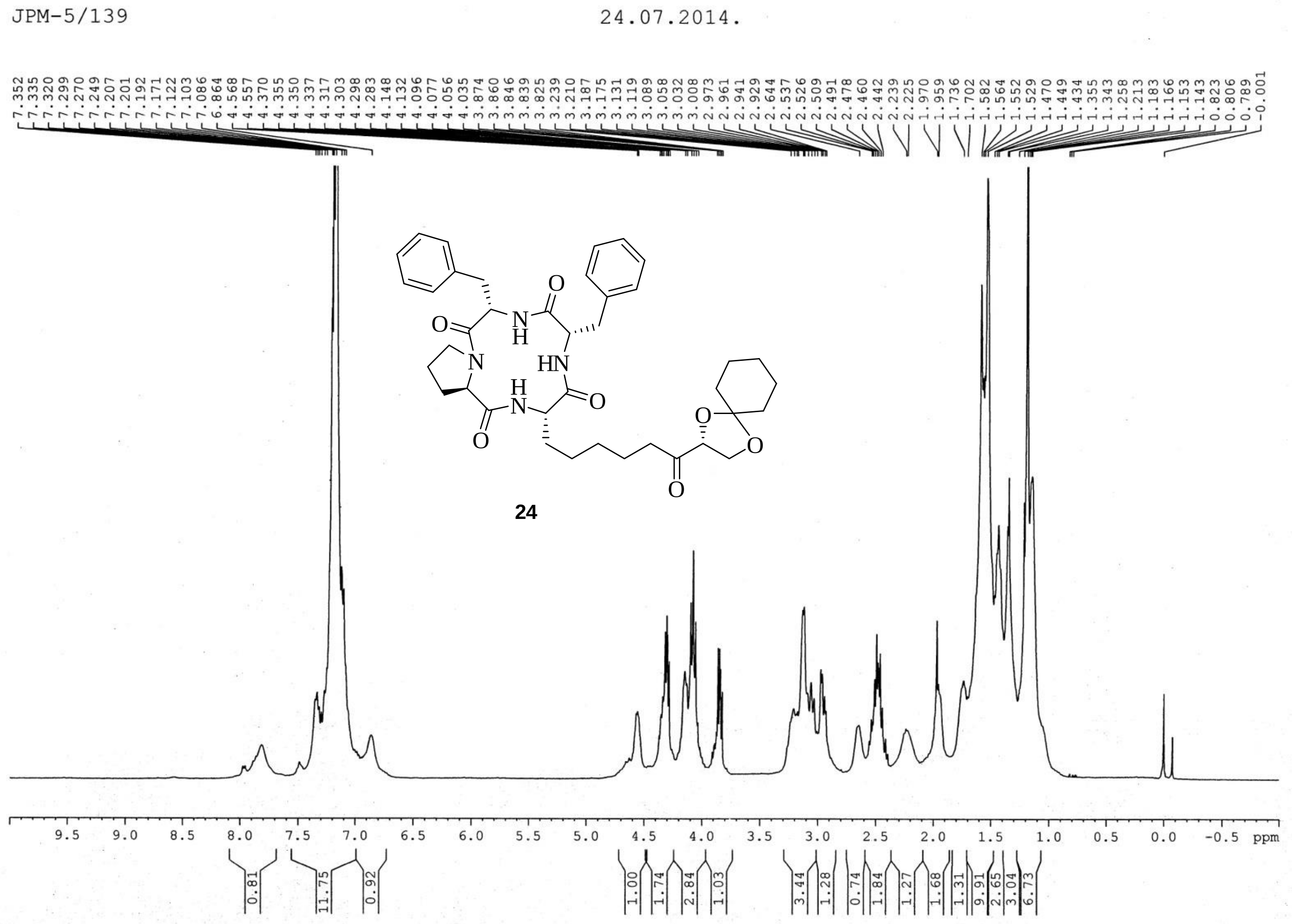

^{1}H NMR spectrum of compound **24** in $CDCl_3$.

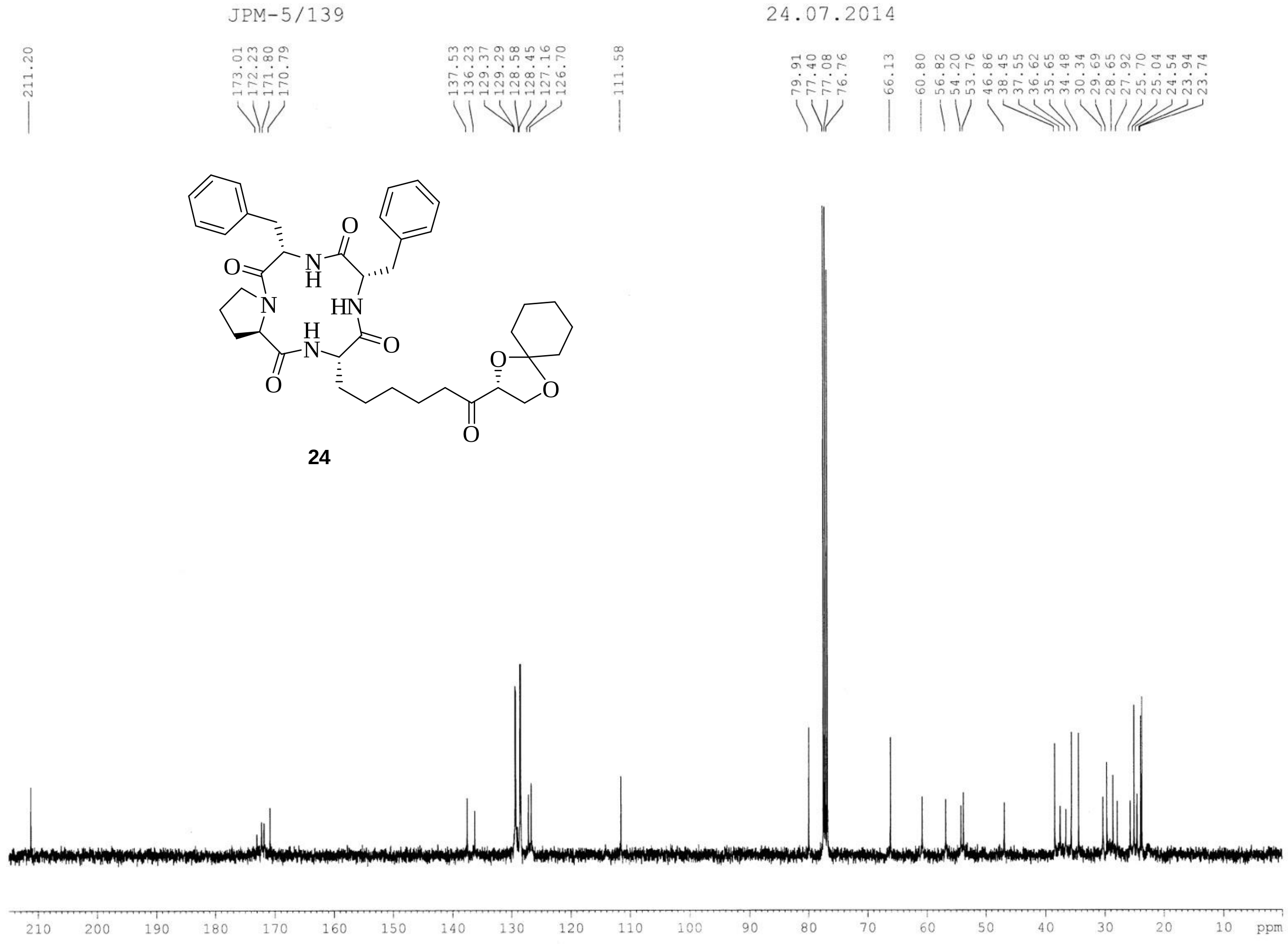

^{13}C NMR spectrum of compound **24** in $CDCl_3$.

JPM-5/140 28.07.2014

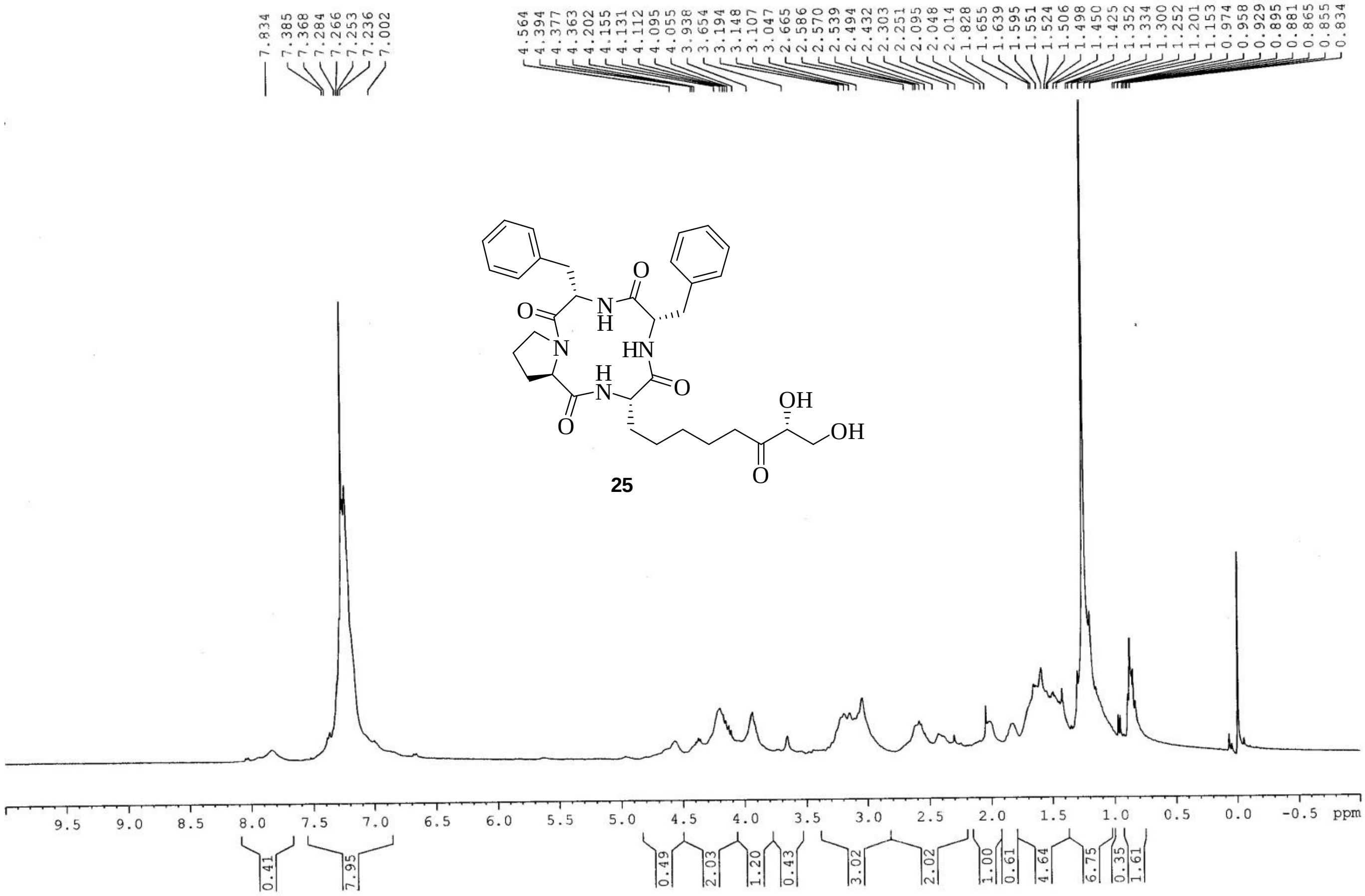

^{1}H NMR spectrum of compound **25** in $CDCl_3$.

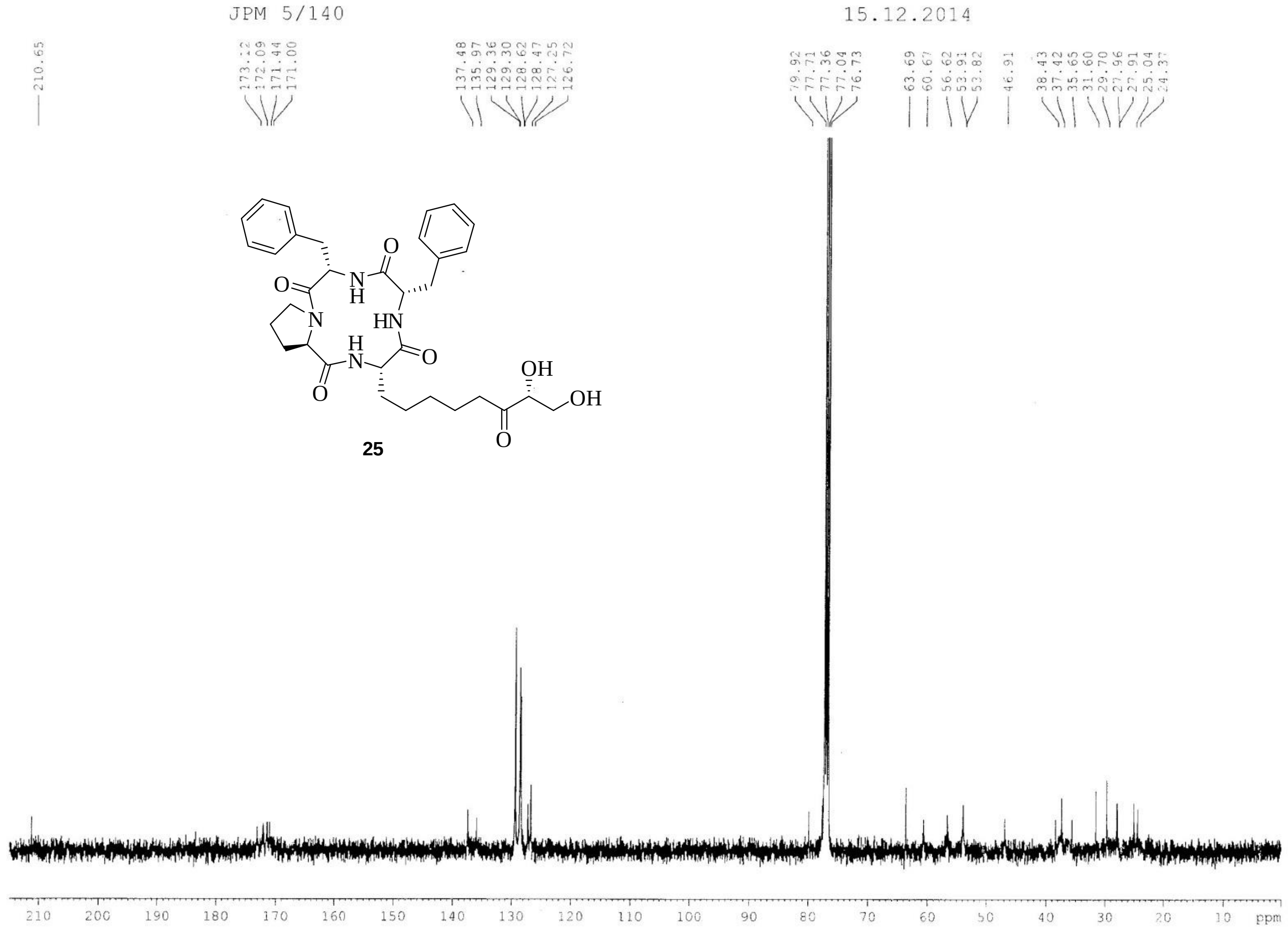

^{13}C NMR spectrum of compound **25** in $CDCl_3$.

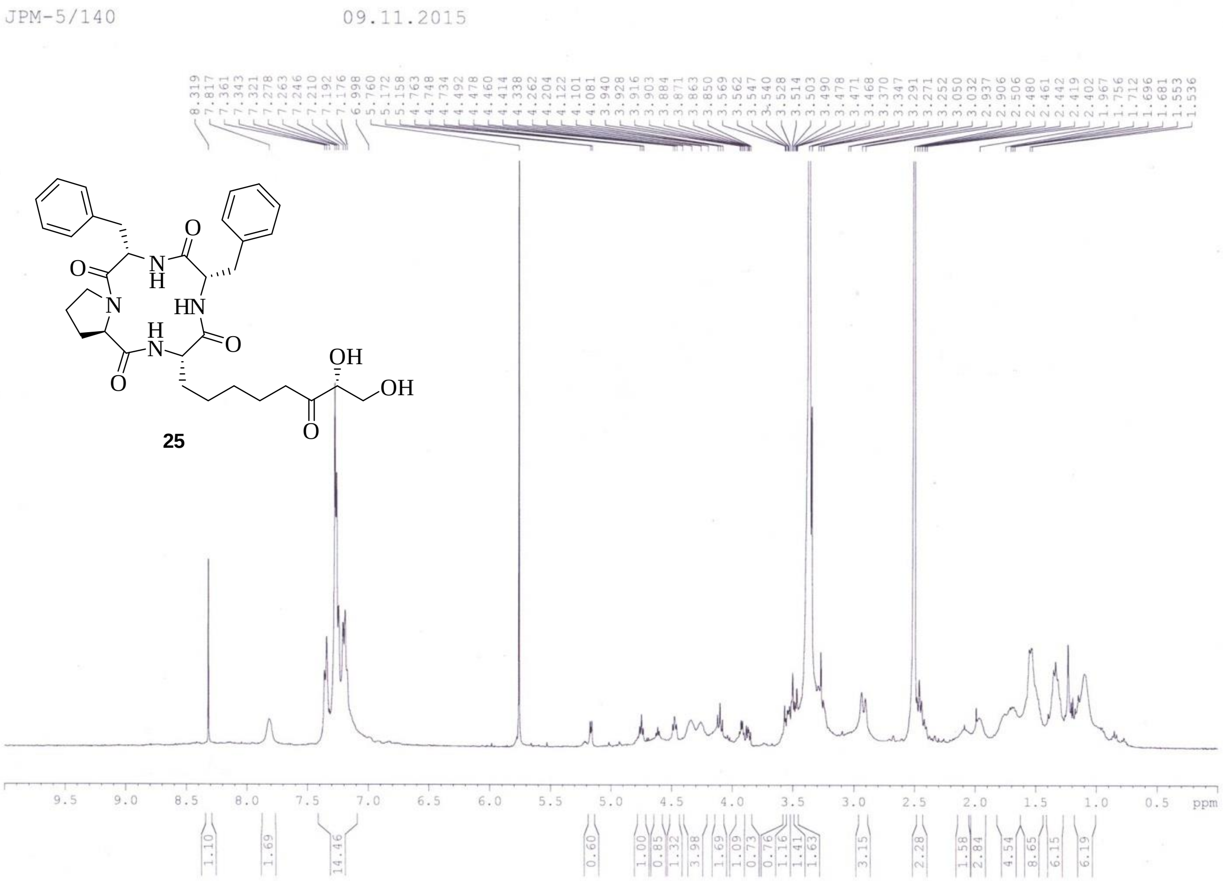

^{1}H NMR spectrum of compound **25** in DMSO-d_6 at 400 MHz.

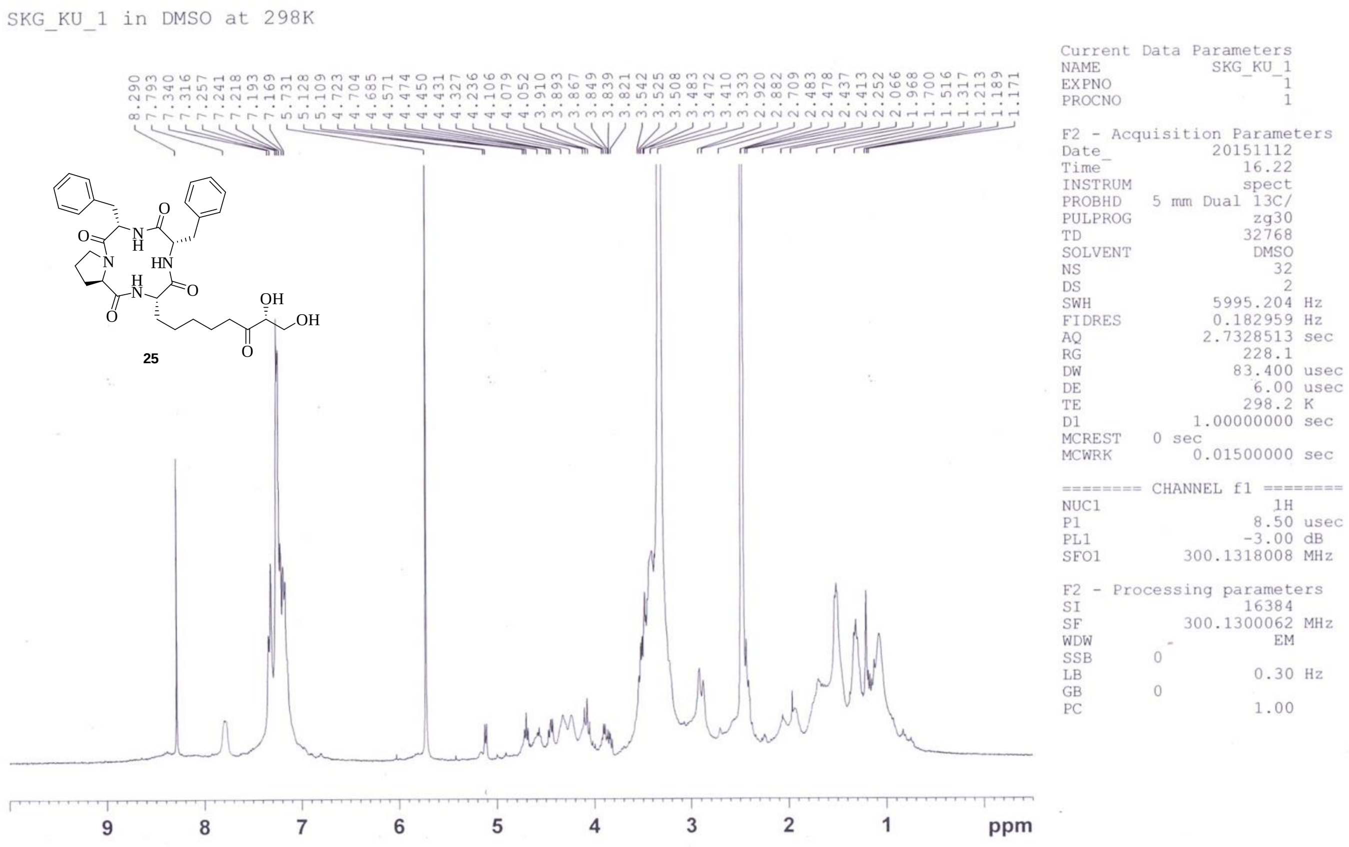

^{1}H NMR spectrum of compound **25** in DMSO-d_6 at 600 MHz at 298 K.

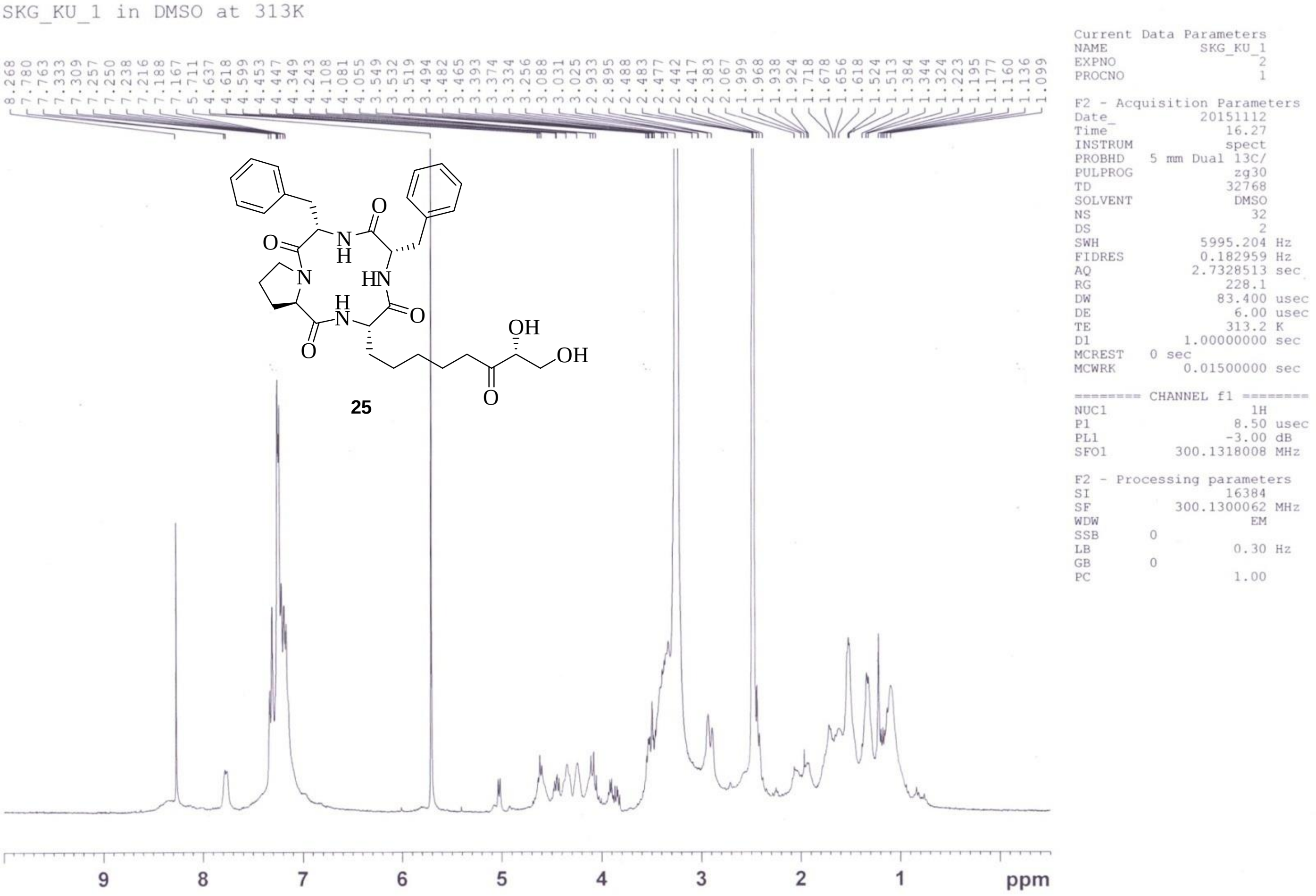

^{1}H NMR spectrum of compound **25** in DMSO-d_6 at 600 MHz at 313 K.

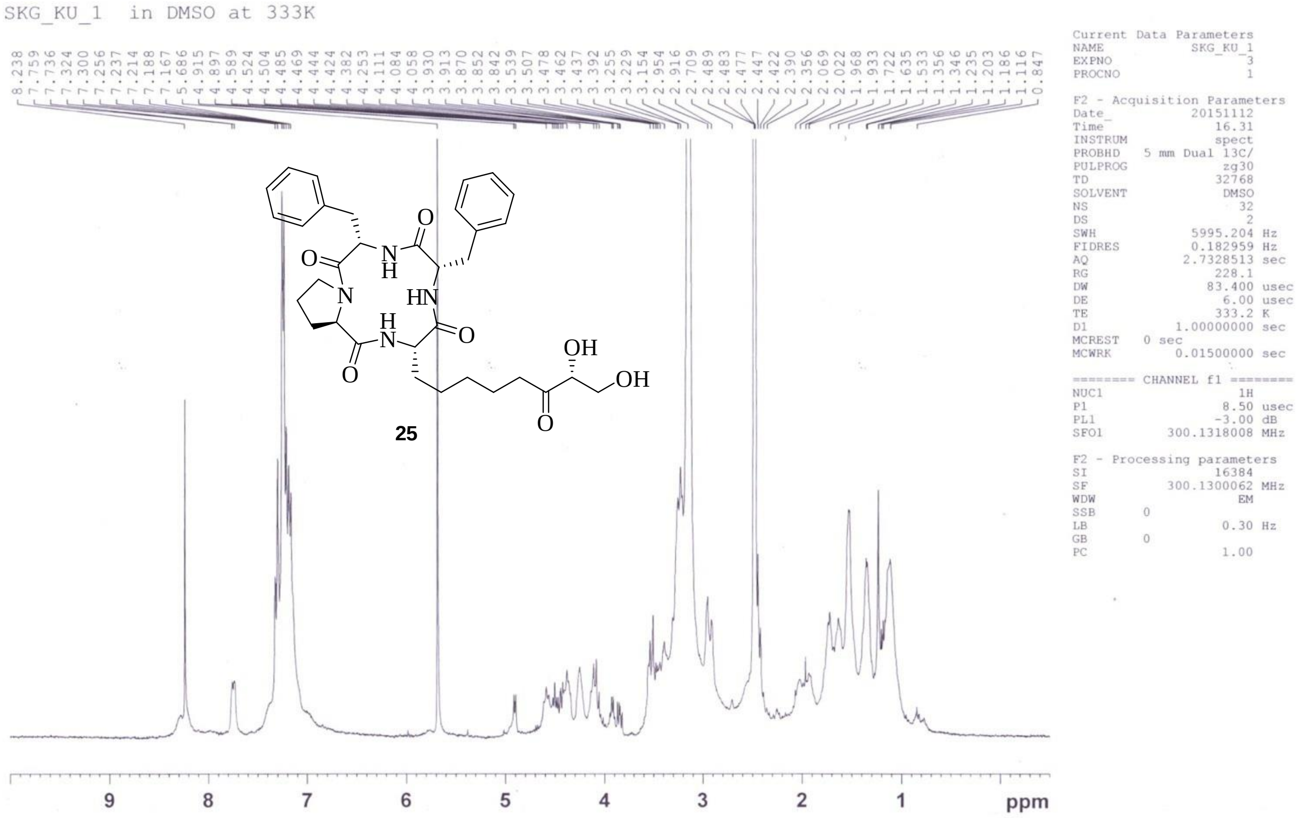

^{1}H NMR spectrum of compound **25** in DMSO-d_6 at 600 MHz at 333 K.

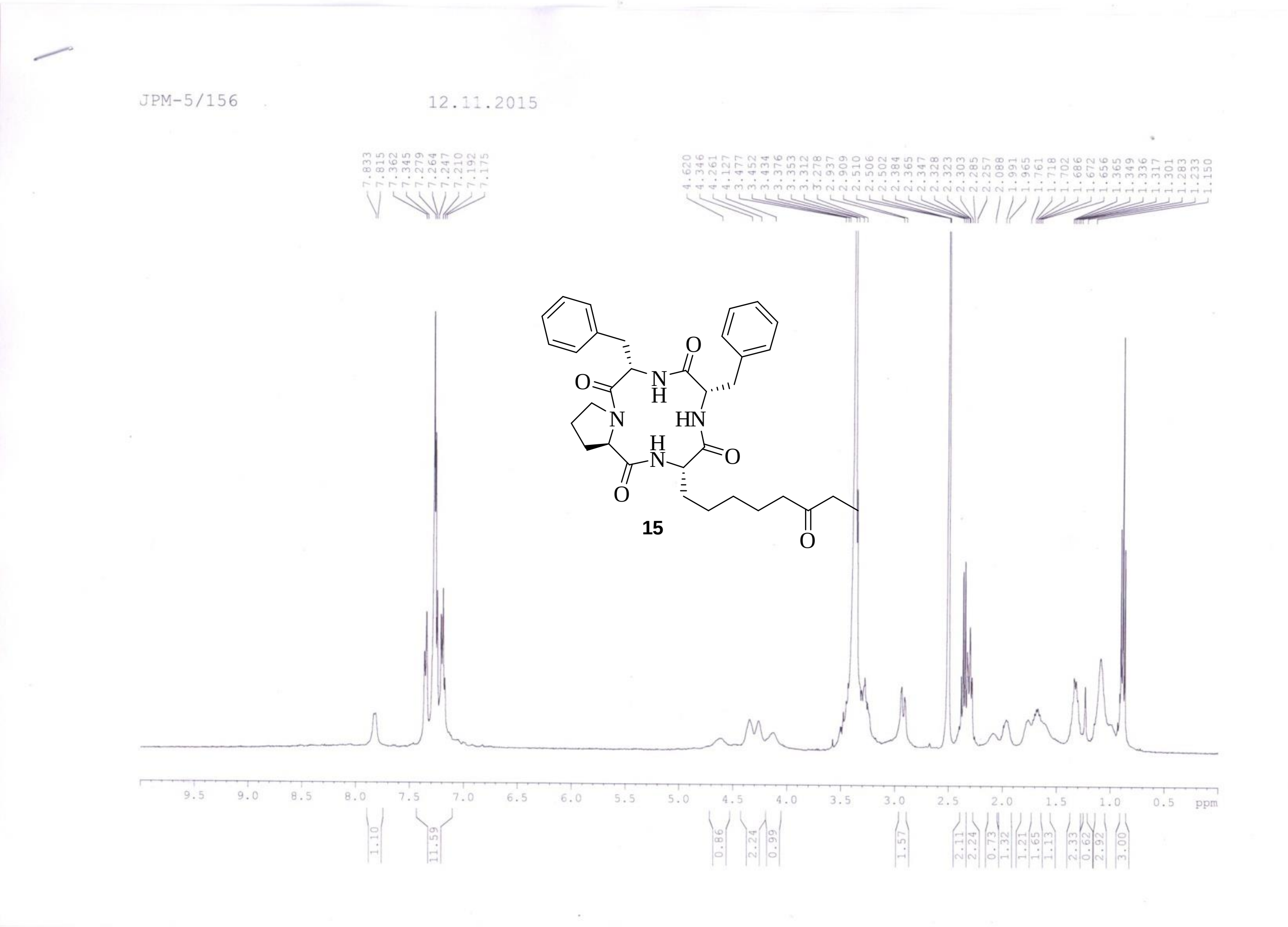

^{1}H NMR spectrum of compound **15** in DMSO-d_6 at 400 MHz at 298 K.

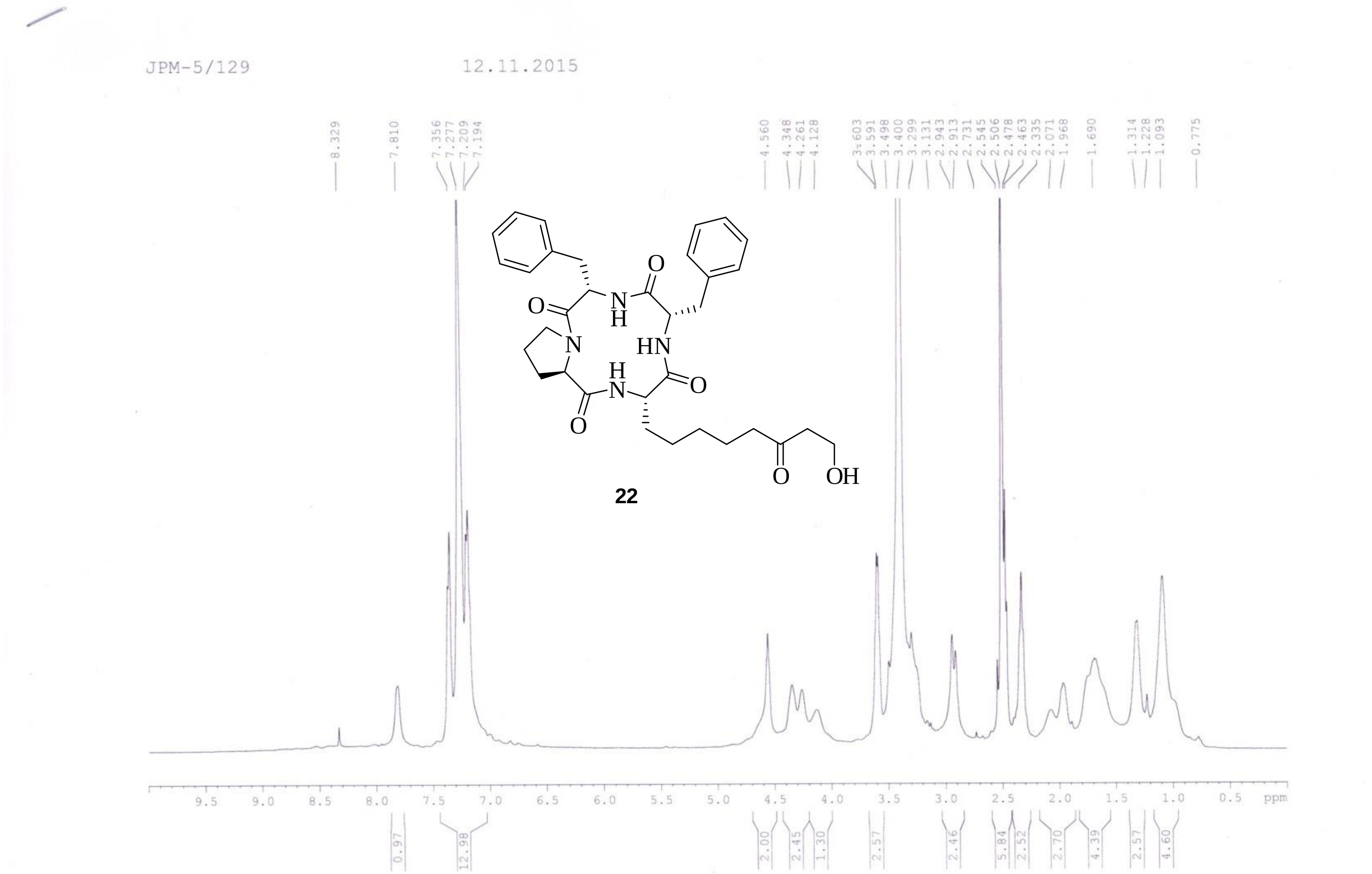

^{1}H NMR spectrum of compound **22** in DMSO-d_6 at 400 MHz at 298 K.
